# Anti-tumor effects of RTX-240: an engineered red blood cell expressing 4-1BB ligand and interleukin-15

**DOI:** 10.1007/s00262-021-03001-7

**Published:** 2021-07-09

**Authors:** Shannon L. McArdel, Anne-Sophie Dugast, Maegan E. Hoover, Arjun Bollampalli, Enping Hong, Zafira Castano, Shannon Curtis Leonard, Sneha Pawar, Jennifer Mellen, Kelvin Muriuki, Douglas C. McLaughlin, Nicholas Bayhi, Christopher L. Carpenter, Laurence A. Turka, Thomas J. Wickham, Sivan Elloul

**Affiliations:** Rubius Therapeutics® Inc., Cambridge, MA USA

**Keywords:** Immunotherapy, CD8-Positive T-Lymphocytes, Cell engineering, Natural killer cells, Investigational therapies

## Abstract

Recombinant agonists that activate co-stimulatory and cytokine receptors have shown limited clinical anticancer utility, potentially due to narrow therapeutic windows, the need for coordinated activation of co-stimulatory and cytokine pathways and the failure of agonistic antibodies to recapitulate signaling by endogenous ligands. RTX-240 is a genetically engineered red blood cell expressing 4-1BBL and IL-15/IL-15Rα fusion (IL-15TP). RTX-240 is designed to potently and simultaneously stimulate the 4-1BB and IL-15 pathways, thereby activating and expanding T cells and NK cells, while potentially offering an improved safety profile through restricted biodistribution. We assessed the ability of RTX-240 to expand and activate T cells and NK cells and evaluated the in vivo efficacy, pharmacodynamics and tolerability using murine models. Treatment of PBMCs with RTX-240 induced T cell and NK cell activation and proliferation. In vivo studies using mRBC-240, a mouse surrogate for RTX-240, revealed biodistribution predominantly to the red pulp of the spleen, leading to CD8 + T cell and NK cell expansion. mRBC-240 was efficacious in a B16-F10 melanoma model and led to increased NK cell infiltration into the lungs. mRBC-240 significantly inhibited CT26 tumor growth, in association with an increase in tumor-infiltrating proliferating and cytotoxic CD8 + T cells. mRBC-240 was tolerated and showed no evidence of hepatic injury at the highest feasible dose, compared with a 4-1BB agonistic antibody. RTX-240 promotes T cell and NK cell activity in preclinical models and shows efficacy and an improved safety profile. Based on these data, RTX-240 is now being evaluated in a clinical trial.

**Supplementary Information:**

The online version contains supplementary material available at 10.1007/s00262-021-03001-7.

## Introduction

Immunotherapy has revolutionized cancer treatment. Checkpoint blockade and T cell therapies have significantly improved survival rates in many diseases [1, 2]. However, most cancers do not respond to checkpoint inhibitors and many patients do not benefit from these approaches. Even among responders, significant toxicities limit their use in many instances [3, 4], highlighting the need for novel immune-focused approaches.

T cells and natural killer (NK) cells have fundamental roles in eliminating cancer [5]. Multiple approaches focus on stimulating the 4-1BB and interleukin 15 (IL-15) pathways to activate and expand anti-tumor T cells or NK cells. IL-15 induces 4-1BB expression on memory CD8 + T cells in an antigen-independent manner and both act cooperatively to promote memory CD8 + T cell responses and survival [6–8]. A fusion protein of IL-15 and 4-1BBL induced T cell proliferation and interferon-γ (IFNγ) secretion from T cells and was more efficacious in a murine lung metastasis model than either protein alone [9]. Preclinical studies with the IL-15 superagonist ALT-803 demonstrated the ability of IL-15–IL-15 receptor α (IL-15Rα) complexes to expand and activate human NK cells and T cells [10], as well as to enhance NK-mediated cytotoxicity toward K562 targets in vitro [11]. Furthermore, co-expression of membrane-presented IL-15 and 4-1BBL on K562 cells enhanced NK cell expansion in vitro [12, 13].

A conventional therapeutic approach, using intravenous (IV) injection of 4-1BB agonist antibodies to expand and activate T cells, has been tested in early phase clinical trials [14, 15]. However, the potential of 4-1BB agonist antibodies is limited due to severe liver toxicity [16, 17] and anti-drug antibodies [15]. An alternative strategy has been to expand NK cells ex vivo for subsequent infusion into patients. This method involves IL-15 and 4-1BBL proteins expressed on the surface of K562 cells, which expands autologous NK cells from peripheral blood in sufficient numbers for potential therapeutic use [12, 13, 18]. However, this requires an ex vivo, personalized 10-day laboratory expansion and subsequent depletion of T cells and the K562–IL-15–4-1BBL cells prior to infusion to avoid toxicity [13].

Engineered human red blood cells (RBCs) that express biotherapeutic proteins within, or on, their cell surface, herein termed Red Cell Therapeutics™ or RCTs™, are allogeneic cellular medicines potentially able to treat a range of diseases. RBCs have been used safely in transfusion medicine for many years and preclinical studies demonstrate that RBCs engineered to present antibody-derived fragments can provide some therapeutic benefit in mice [19, 20]. Thus, we evaluated RBC engineering as a novel approach to induce T and NK cell immune activation. Crucially, RBC distribution is limited to the vascular system, potentially minimizing off-tissue toxicity.

To investigate the potential of genetically engineered RBCs to induce immune-cell activation and promote anti-tumor immunity, we created RTX™-240. RTX-240 is an allogeneic, engineered, enucleated, human RBC with cell-surface expression of 4-1BBL and trans-presented IL-15 (IL-15TP), a fusion of IL-15 and IL-15Rα. Here, we show that RTX-240 promotes NK cell and T cell expansion and activation in vitro and exhibits the synergistic effects of combining 4-1BB and IL-15 pathways. A mouse surrogate, mRBC-240, activated T cells and NK cells in vivo, leading to reduction in tumor growth in two different models (B16-F10 and CT26). Importantly, mRBC-240 was primarily distributed to the spleen and was tolerated at the highest feasible dose, compared with a 4-1BB agonistic antibody. Together, our data demonstrate the potential therapeutic effect of RTX-240 in the treatment of cancer.

## Materials and methods

### Erythroid cell engineering

In vitro generation of human engineered RBCs from hematopoietic progenitor cells was performed as previously described [20], with modifications, using lentiviral vectors to induce stable expression of wild-type or engineered proteins [21, 22].

Briefly, human CD34 + hematopoietic progenitor cells (HemaCare, Inc.) from normal human donors were transduced with lentiviral vectors to express OX40L (RCT-OX40L-HA), GITRL (RCT-GITRL-HA), 4-1BBL (RCT-4-1BBL or RCT-4-1BBL-HA), IL-15TP (RCT-IL-15TP) or both 4-1BBL and IL-15TP (RTX-240). Un-transduced cells were used as control (RCT-CTRL). The gene encoding the protein of interest was cloned into the multiple cloning site of a lentiviral vector under the control of the MSCV promoter sequence. Lentivirus was produced in 293 T cells (American Type Culture Collection, ATCC®). The fusion proteins consisted of: glycophorin A (GPA), human IL-15 and the extracellular portion of human IL-15Rα; GPA and the extracellular portion of human 4-1BBL; GPA, the extracellular portion of either human 4-1BBL or OX40L or GITRL and HA tag. Cells were cultured in expansion, differentiation and maturation conditions, using different compositions of growth factors and cytokines based on established erythrocyte culture protocols with modifications. Cells were collected, washed, and counted by flow cytometry before use in downstream assays. Successful generation of RCTs was assessed by expression and ligand-binding assays using flow cytometry.

Mouse surrogate RBCs were engineered using the principles of click chemistry bioconjugation [23]. This method uses the specific reaction between an azide group provided by the 6-azidohexanoic acid sulfo-N-hydroxysuccinimidyl (NHS) ester and a dibenzocyclooctyne (DBCO) group moiety in order to generate mRBCs conjugated with recombinant proteins. Recombinant murine 4-1BBL (m41BBL) and Fc-human IL-15/IL-15Rα fusion (IL-15TP) were produced in Expi293F™ (Thermo Fisher Scientific), purified and concentrated to > 1 mg/mL prior to labeling. Endotoxin measurements in protein preparations were less than 10 endotoxin units (EU)/mg. Following production and purification, m4-1BBL and IL-15TP were conjugated to a DBCO group moiety, which reacts with the azide group in the click reaction. mRBCs obtained from whole blood were filtered to remove leukocytes and labeled with 6-azidohexanoic acid NHS ester. The labeled mRBCs were chemically conjugated with either DBCO-labeled m4-1BBL (mRBC-4-1BBL), DBCO-labeled IL-15TP (mRBC-IL-15TP), or DBCO-labeled m4-1BBL and IL-15TP (mRBC-240) during an overnight incubation. mRBC-CTRL was prepared similarly to mRBC-240 without the addition of any DBCO-labeled protein. Conjugated cells were washed with phosphate-buffered saline (PBS) and stained with anti-mouse 4-1BBL or anti-mouse IgG2a antibodies and analyzed via flow cytometry to determine the protein labeling efficiency.

### Expression and ligand-binding assays

For RCTs, ligand-binding assays were conducted by incubating the RCTs with His-tagged recombinant receptors in PBS at room temperature or on ice for 30 min, then staining for ligands (anti-HA, GG8-1F3.3.1, Miltenyi Biotec, Inc.) and receptors (anti-His, AD1.1.10, Abcam® plc.). In some cases, RCTs were directly stained for 4-1BBL and IL-15. Receptor binding was measured by flow cytometry.

For mRBC, 5 × 10^6^ mRBCs were incubated with 1 μg of recombinant mouse 4-1BB human Fc chimera protein (R&D Systems™, Inc.) for 30 min. Following binding, staining was performed with anti-mouse IgG2a antibody (RMG2a-62, BioLegend®) to detect mRBC-240 and anti-human Fc antibody (HP6017, BioLegend) to detect recombinant m4-1BB. Cells were run on a NovoCyte® 3000 flow cytometer (ACEA Biosciences™, Inc.), and binding was determined by analyzing the percentage of double positive events using FlowJo™ software (BD Biosciences™, Inc.).

### In vitro* reporter assays*

#### Human reporter assays

To assess human TNF-family receptor activation, NFkB reporter Jurkat cells expressing GITR, OX40 or 4-1BB (GITR Bioassay, OX40 Bioassay, 4-1BB Bioassay, respectively, Promega® Corporation) were used according to the manufacturer’s instructions. Briefly, reporter cells were thawed, added to opaque assay plates in RPMI with 1% fetal bovine serum (FBS) and incubated with engineered RBCs (starting at 2 × 10^5^ per well) or controls, for 6 h at 37 °C. NFkB activation was measured by luminescence using the Bio-Glo™ Luciferase Assay System (Promega Corporation) on a multi-mode plate reader. Fold change in luminescence was calculated by dividing the signal of each test sample by the average of the negative controls (reporter cells and media only).

Human 4-1BBL activity was measured using the 4-1BB/NFkB reporter HEK293 cell line (BPS Bioscience, Inc.), maintained according to the vendor’s instructions and used between passages 4 and 20. For reporter assays, 3.5 × 10^4^ cells were cultured overnight at 37 °C in assay media (DMEM supplemented with 10% FBS, 1% penicillin/streptomycin, 1% MEM NEAA, 1 mM sodium pyruvate) in opaque assay plates, then incubated with engineered RBCs (starting at 2 × 10^5^ cells per well) or controls (4-1BB agonistic antibody at 10 nM or 1 nM plus F(ab’)2-Goat anti-human IgG Fc gamma secondary antibody [Thermo Fisher Scientific] at 25 nM or 2.5 nM) at 37 °C for 6 h. NFkB activation was measured by luminescence (ONE-Step™ Luciferase Assay System, BPS Bioscience) on a multi-mode plate reader. Fold change in luminescence was calculated by dividing the signal of each test sample by the average of the negative controls (reporter cells and media only).

Functionality of IL-15TP was determined using the HEK-Blue™ IL-2 reporter cell line (InvivoGen®), which expresses the IL-2Rβ and IL-2Rγ receptors common to both IL-2 and IL-15. HEK-Blue IL-2 reporter cells were maintained according to the vendor’s instructions and used between passages 4 and 20. Serial dilutions of RCTs or mRBCs were prepared starting at 1.3 × 10^5^ for RCT and 1 × 10^7^ cells per well for mRBC and incubated in assay media (DMEM with 4.5 g/L glucose + 2 mM L-glutamine + 10% FBS + 1% penicillin/streptomycin + 1 μg/mL Normocin™ antimicrobial reagent) in 96-well tissue-culture treated plates with 5 × 10^4^ HEK-Blue IL-2 cells for 20 h at 37 °C. Supernatants were collected and secreted embryonic alkaline phosphatase (SEAP) was quantified using QUANTI-Blue™ Solution (InvivoGen) according to the manufacturer’s instructions. Optical density was read at 655 nM using a microplate reader (SpectraMax® M3 microplate reader, Molecular Devices®, LLC). Fold change in SEAP secretion was calculated by dividing the concentration in each test well by the average of the concentration of the negative controls (reporter cells, media only, RCT-CTRL or mRBC-CTRL).

#### Murine reporter assays

Assessment of m4-1BB activation and downstream signaling was performed using a mouse 4-1BB/NFkB reporter cell line. Serial dilutions of mRBC-CTRL or mRBC-240 were prepared starting at 1 × 10^7^ cells/well and incubated with 2 × 10^4^ m4-1BB/NFkB reporter cells for 24 h at 37 °C. The Bio-Glo Luciferase Assay System was used and luminescence was measured on a Synergy™ HTX multi-mode microplate reader (BioTek® Instruments, Inc.). The fold change of mRBC-240 luminescence over that of mRBC-CTRL was reported.

Assessment of IL-15 activity on mRBC was performed using the HEK-Blue IL-2 reporter cell line assay (detailed in human reporter assays).

### Primary-cell assays

Cryopreserved PBMCs from human donors were thawed and washed, then labeled with CellTrace™ Far Red dye (CTFR, Thermo Fisher Scientific) according to the manufacturer’s instructions. RCTs were collected and washed. 1–2 × 10^5^ PBMCs were added to 96-well round-bottom plates along with RCTs (1.25–5 × 10^5^) or control treatments of rIL-15 (PeproTech®, Inc.) at 1 or 0.1 ng/mL or 4-1BB agonistic antibody at 10 nM or 1 nM plus F(ab’)2-Goat anti-human IgG Fc gamma secondary antibody at 25 nM or 2.5 nM. In some assays, purified anti-CD3 antibody (OKT3, Thermo Fisher Scientific) was added to a final concentration of 0.5 μg/mL. Plates were incubated at 37 °C for 5–8 days before staining for flow cytometry (detailed in immune profiling).

Purified NK cells were thawed, washed, and added to a 96-well round-bottom plate in assay media with RCTs (1:5 NK:RCT ratio) or control treatments and incubated overnight at 37 °C. K562 cells (CCL-243, ATCC) were labeled with CTFR according to the manufacturer’s instructions, added to the pre-activated NK cells or control wells and incubated at 37 °C for 4 h. To measure target cell killing, samples were stained with LIVE/DEAD™ Fixable Aqua Dead Cell Stain (LD, Thermo Fisher Scientific) and washed and live K562 cells were enumerated by flow cytometry as CTFR-positive, LD-negative cells. Specific killing was calculated as percentage live targets with no NK minus percentage live targets with NK for each control and treatment condition.

To assess mRBC effects on T cells and NK cells, single-cell suspensions were prepared from the spleens of C57BL/6 wild-type mice and 1.5 × 10^5^ splenocytes were incubated with 1 × 10^6^ mRBCs in the presence of 1 μg/mL anti-CD3 antibody (145-2C11, Thermo Fisher Scientific) for 4 days at 37 °C. Cells were collected, and the staining of CD8 + T cells and NK cells for flow cytometry was performed using anti-mouse CD16/CD32 antibody (BioLegend), LD Fixable Aqua Dead Cell Stain and fluorophore-conjugated antibodies recognizing the extracellular CD8 (5.3–6.7, BD Bioscience) and NK1.1 (PK136, BioLegend) proteins. Data were acquired on a BD LSRFortessa™ flow cytometer (BD Biosciences) and analyzed with FlowJo software. To assess IFNγ production, single-cell suspensions were prepared from spleens of C57BL/6 wild-type mice and 1.5 × 10^5^ splenocytes were incubated with serial dilutions of mRBCs starting at 1 × 10^7^ cells in the presence of 2 μg/mL of anti-CD3 antibody (145-2C11, Thermo Fisher Scientific) for 24 h at 37 °C. Supernatants were collected and secreted IFNγ was measured using the BD OptEIA™ Mouse IFNγ ELISA Set (AN-18, BD Bioscience) according to the manufacturer’s instructions. Optical density was read at 450 nM using a microplate reader.

### Immune-cell profiling

Details of the antibodies used and gating strategies for the identification of cell populations are provided in the supplementary tables 1 and 2. Staining of immune cells for flow cytometry was performed using a mix of fluorophore-conjugated antibodies and either LD Fixable Aqua Dead Cell Stain or Zombie NIR™ dye (BioLegend) in PBS. Fc receptors were blocked with anti-mouse CD16/CD32 antibody (BioLegend) for mouse staining. For intracellular staining, cells were fixed with either 4% paraformaldehyde (Electron Microscopy Sciences) or Foxp3 Fix/Perm fixation buffer (Foxp3/Transcription Factor Staining Buffer Set, eBioscience), washed with a permeabilization buffer, then stained for intracellular antigens in a Foxp3 Fix/Perm permeabilization buffer (eBioscience). After this, cells were washed with a permeabilization buffer and resuspended in PBS for analysis by flow cytometry according to the manufacturer’s instructions. Data were acquired on a BD™ LSRFortessa™ flow cytometer (BD Biosciences) or a NovoCyte 3000 flow cytometer and analyzed with FlowJo software.

### In vivo* activity of engineered RBCs*

Female C57BL/6 or BALB/c mice 6–8 weeks old (Charles River Laboratories or The Jackson Laboratory) were housed in IACUC-accredited animal facilities under specific pathogen-free conditions. Protocols were approved by IACUC and studies complied with ethical regulations and humane endpoints.

#### B16-F10 metastatic model

The murine melanoma cell line B16-F10 (ATCC) was cultured according to ATCC recommendations and 1 × 10^5^ cells were injected IV in 200 μL RPMI-1640 into C57BL/6 mice to establish a pulmonary metastatic melanoma model. On days 1, 5, and 8 post-inoculation, animals received either 1 × 10^9^ mRBCs IV, 4-1BB agonistic antibody (InVivoMab™ anti-mouse 4-1BB clone 3H3, Bio X Cell, Inc., 2.5 mg/kg) intraperitoneally (IP), or rIL-15 (0.2 mg/kg) IP. On day 14, animals were euthanized and lungs were perfused with ice-cold PBS through the right atrium prior to harvest. The left lobe was collected into RPMI-1640 for immune phenotyping by flow cytometry. Remaining tissue was fixed in 10% buffered formalin for 24 h, switched to 70% ethanol and metastases enumerated under the microscope. For evaluation of frequency of immune cells in the spleen, organs were harvested on day 12 and CD8 + T cells and NK cells were analyzed by flow cytometry.

#### CT26 subcutaneous model

The murine colon cancer cell line CT26 (ATCC) was cultured according to ATCC recommendations and 1 × 10^5^ cells were implanted subcutaneously in 100 μL RPMI-1640 + 50% Matrigel® matrix (Corning) into the right flank of BALB/c mice. When tumors reached 80–120 mm^3^, mice were randomized and treated IV with 1 × 10^9^ of either mRBC-240 or mRBC-CTRL on days 1, 5, and 8 post-randomization. Tumor volume was measured three times a week and calculated as length (mm) × width^2^ (mm) × 0.5. On day 11, animals were euthanized and tumors harvested for immune phenotyping by flow cytometry.

### *mRBC labeling for *in vivo* detection*

To detect chemically conjugated mRBCs in circulation, 2 × 10^9^/mL mRBCs were incubated with 1 μM DBCO-conjugated cyanine5 (Cy5, Click Chemistry Tools, LLC) for 20 min in the dark at room temperature. For histology, 1 × 10^9^/mL mRBCs were labeled with 10 μM CTFR for 6 min in the dark, quenched with FBS and washed prior to formulation.

### Pharmacokinetics (PK) of mRBC-240

The percentage of Cy5-labeled mRBC in C57BL/6-naïve mice was evaluated using flow cytometry. Mice were dosed IV with mRBC-CTRL or mRBC-240 (at 2 dose levels) on days 0, 3, 7, and 10. Whole blood was collected into blood collection tubes with K_2_EDTA by tail-nick bleed 4 h following the first mRBC dose and then daily until day 14. Samples were run on a NovoCyte® 3000 flow cytometer (Acea Biosciences™, Inc.; 100,000 events/sample), and the percentage of Cy5-positive singlet cells was analyzed using FlowJo™ software (version 10, BD Biosciences).

### Biodistribution of mRBC-240 in the CT26 tumor model

1 × 10^9^ CTFR-labeled mRBC-CTRL or mRBC-240 were injected IV in CT26 tumor-bearing mice. One day after dosing, tissues were collected from mice (without perfusion) and frozen in Tissue-Tek® Cryomold® plastic molds (VWR International, LLC) containing Tissue-Tek O.C.T. Compound (VWR International, LLC). Tissue blocks were sectioned on a Leica® CM1520 cryostat (Leica Microsystems Inc.) and mounted onto slides for staining. Slides were fixed in 4% formalin, blocked with Background Sniper blocking reagent (Biocare Medical, LLC) and staining was performed with anti-CD31 antibody (clone 2H8, MilliporeSigma), anti-hamster Alexa Fluor® 488 dye-conjugated secondary antibody (Jackson ImmunoResearch Laboratories, Inc.) and Hoechst dye. Scanning was performed on the Aperio™ ScanScope® FL scanner (Leica Microsystems Inc.), and images were analyzed using the HALO image analysis software module High Plex FL V2.0 (Indica Labs) to quantify density (cells per mm^2^) of mRBC-240 and mRBC-CTRL in each tissue.

### *Pharmacodynamics (PD) and *in vivo* tolerability of mRBCs*

Blood was collected by cardiac puncture in BD Microtainer® blood collection tubes with K_2_EDTA tubes (BD Biosciences) and single-cell suspensions were prepared via treatment with Alfa Aesar™ RBC Lysis Buffer for mouse RBCs (Thermo Fisher Scientific) according to the manufacturer’s instructions. Spleens were processed into single-cell suspensions by mechanical dissociation through a Corning® 70 μm Cell Strainer (Corning, Inc.) and subsequent lysis of RBCs with ACK Lysing Buffer (Thermo Fisher Scientific). Single-cell suspensions from tumors, lungs and livers were prepared using the murine Tumor Dissociation Kit, the murine Lung Dissociation Kit, or the murine Liver Dissociation Kit (Miltenyi Biotec), respectively, according to the manufacturer’s instructions. For intracellular cytokine staining, single spleen cell suspensions were incubated for 4 h at 37 °C with eBioscience™ Cell Stimulation Cocktail (Thermo Fisher Scientific) before staining according to the immune profiling section.

Toxicity of mRBC-240 in mice was determined using a previously described model [17], with adaptations: mice were administered mRBC-CTRL or mRBC-240 at 3 dose levels (1 × 10^9^, 3 × 10^8^ or 1 × 10^8^) or 4-1BB agonistic antibody (*InVivo*Mab anti-mouse 4-1BB clone 3H3, 10 mg/kg or 2.5 mg/kg) on days 1, 5, 8, and 11. On day 18, animals were euthanized and livers fixed in 10% buffered formalin for 24 h and paraffin embedded. Tissue sections were either stained with hematoxylin and eosin (H&E) using standard procedures or with anti-mouse F4/80 antibody (clone BM8, Thermo Fisher Scientific). H&E sections were blind-scored by a pathologist based on necrosis and inflammation in the tissue and vessels. F4/80 staining quantification was performed using HALO image analysis software. For liver enzyme analysis, whole blood was collected via cardiac puncture and alanine aminotransferase (ALT) levels (U/L) measured in the serum.

### Statistical analysis

A one-way ANOVA was used to account for any differences between groups for data in vitro or in vivo. A two-way ANOVA was used to account for the difference in CT26 tumor growth between mRBC-240 and mRBC-CTRL. A t test was used to compare the density of mRBC-240 and mRBC-CTRL in different organs.

## Results

### RCTs can express functional costimulatory ligands

To evaluate the ability of RCTs to stably express functional immunostimulatory ligands, CD34 + cells were transduced with lentiviral vectors encoding either OX40L, GITRL or 4-1BBL as HA-tagged fusion proteins of GPA. Robust surface expression of each fusion protein was confirmed by flow cytometry, including binding to recombinant His-tagged receptor (supplementary Fig. 1a) and ligand function was demonstrated through dose-dependent induction of NFkB activity (supplementary Fig. 1b). Cell-surface expression of 4-1BBL led to greater activation of NFkB (supplementary Fig. 1c) and greater proliferation of human CD4 + T cells (supplementary Fig. 1d) and CD8 + T cells (supplementary Fig. 1e) than with the 4-1BB agonistic antibody, indicating that RCT-4-1BBL induces a more robust downstream signaling of the 4-1BB pathway.

### *Characterization and Effects of RTX-240 *in vitro

#### Validation of 4-1BBL and IL-15TP expression on RTX-240

**Fig. 1:**
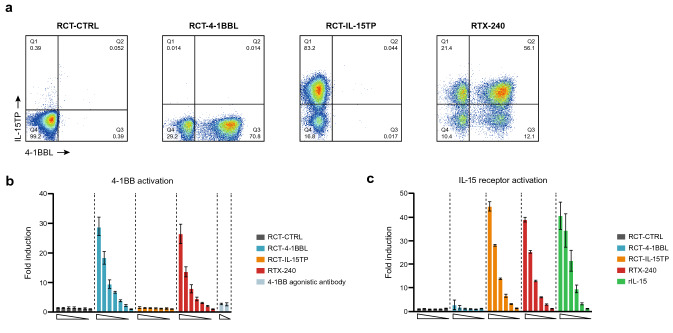
4-1BBL and IL-15TP on RTX-240 stimulate receptor-specific activation in reporter cells. **a** Representative flow cytometry plots of RTX-240 stained for 4-1BBL and IL-15TP. **b** Fold change in NFkB activation in 4-1BB/NFkB reporter HEK293 cells incubated with engineered RBCs or 4-1BB agonistic antibody + cross-linking antibody (10 nM and 1 nM agonist with 25 nM and 2.5 nM cross-linker, respectively) compared with media alone. **c** Fold change in JAK/STAT activation by IL-15 in reporter HEK-Blue-IL-2 cells incubated with engineered RBCs or recombinant (r) IL-15 (threefold dilutions from 100 pg/mL), compared with media alone. Bars indicate SD of 2–3 technical replicates. IL-15TP, trans-presented interleukin 15; RBC, red blood cell; SD, standard deviation

To simultaneously activate key co-stimulatory and cytokine pathways, we developed RTX-240, engineered RBCs which co-express 4-1BBL and IL-15TP on the cell surface. Flow cytometry confirmed that RTX-240 consisted primarily of cells co-expressing 4-1BBL and IL-15TP, with a minority of cells expressing each ligand alone (Fig. 1a). Incubation of RTX-240 or RCT-4-1BBL with 4-1BB–NFkB 293 T-reporter cells resulted in a greater than 25-fold increase in NFkB activation versus media alone (Fig. 1b). Similarly, incubation of RTX-240 or RCT-IL-15TP with IL-2–JAK/STAT reporter cells led to a 45-fold and 39-fold increase (vs media alone) in JAK/STAT pathway activation, respectively (Fig. 1c). These data indicate that RTX-240 delivers 4-1BBL- and IL-15-specific signals that are not compromised by ligand co-expression.

#### *RTX-240 stimulates human T cells *in vitro* in the presence of anti-CD3 stimulation*

**Fig. 2:**
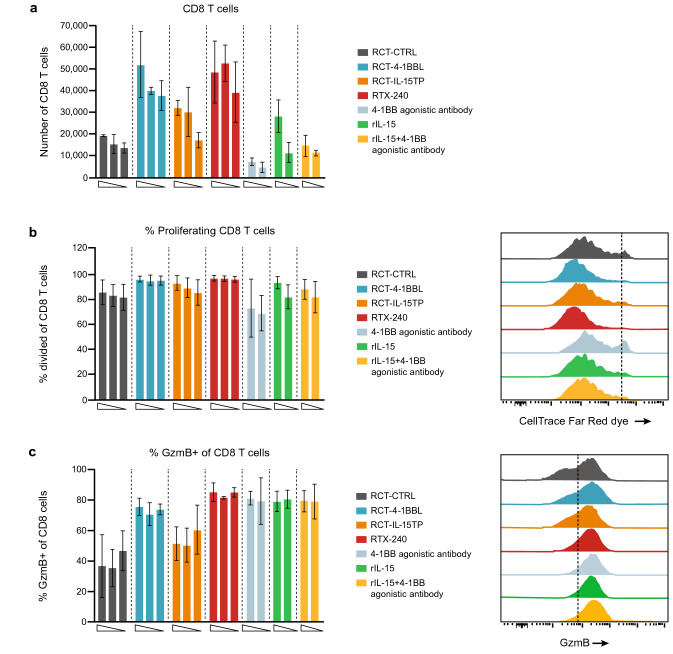
Co-stimulation of human T cells in vitro. PBMCs were labeled with CTFR and incubated with anti-CD3 (0.5 μg/mL) and the indicated treatments for 5 days, then analyzed by flow cytometry for: **a** CD8 + T cell number, **b** CD8 + T cell proliferation (percentage of cells that went through at least one division) and Granzyme B expression on CD8 + T cells **c**. Bars indicate SD of 3 biological replicates. Flow plots are representative data from one donor. CTFR, CellTrace Far Red dye; GzmB, Granzyme B; IL-15TP, trans-presented interleukin 15; PBMC, peripheral blood mononuclear cell; rIL-15, recombinant IL-15; SD, standard deviation

IL-15 and 4-1BBL both promote T cell survival and proliferation [24, 25]. To evaluate the co-stimulatory effects of RTX-240 on human T cells, CTFR-labeled PBMCs were incubated with anti-CD3 antibody, along with RCTs or control treatments. Notably, RCT-4-1BBL, RCT-IL-15TP or RTX-240 led to greater expansion of CD8 + T cells than the combination of rIL-15 plus 4-1BB agonistic antibody (Fig. 2a), thus demonstrating the potency of ligands expressed on a cell surface. While the majority of anti-CD3-treated PBMCs were dividing by day 5, a small increase in CD8 + T cell proliferation was observed with RCT-4-1BBL and RTX-240 (Fig. 2b), suggesting that the increase in CD8 + T cell expansion was due to proliferation and survival. Furthermore, treatment with RTX-240 or RCT-4-1BBL resulted in a twofold increase in the percentage of Granzyme B + CD8 + T cells as compared with RCT-CTRL (Fig. 2c), as with 4-1BB agonistic antibody or rIL-15 treatments.

#### *RTX-240 provides direct stimulation of human memory CD8* + *T cells and NK cells *in vitro

**Fig. 3:**
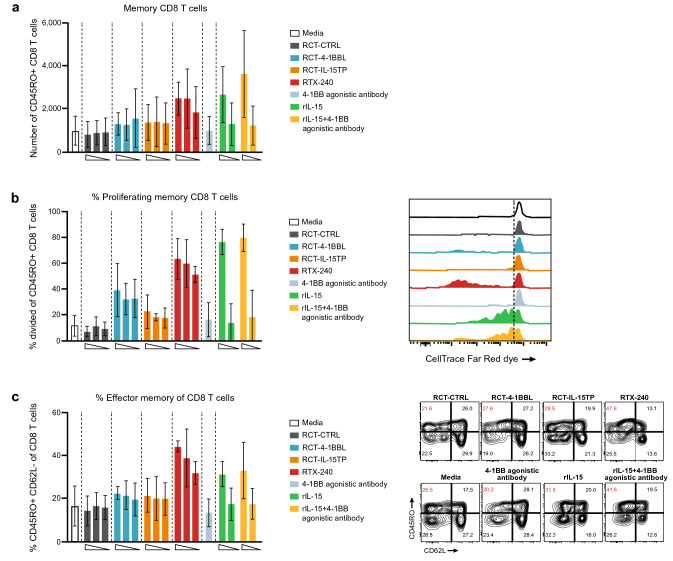

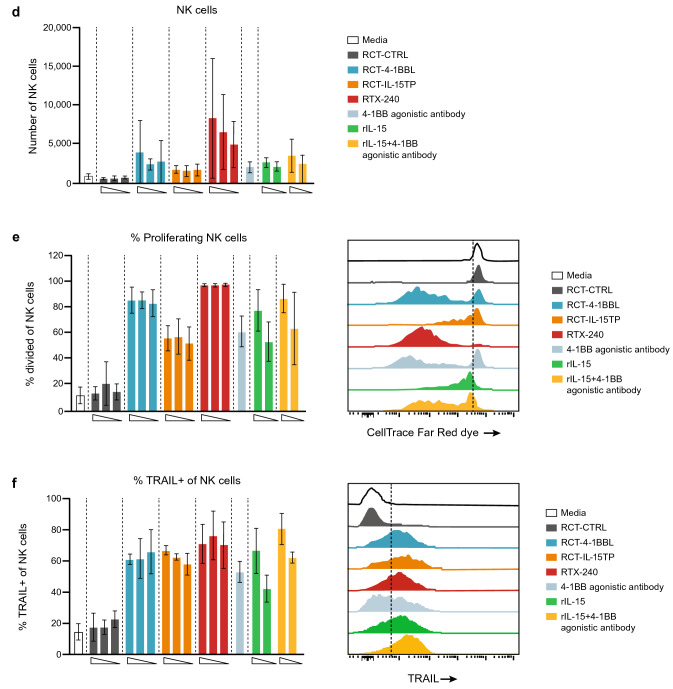

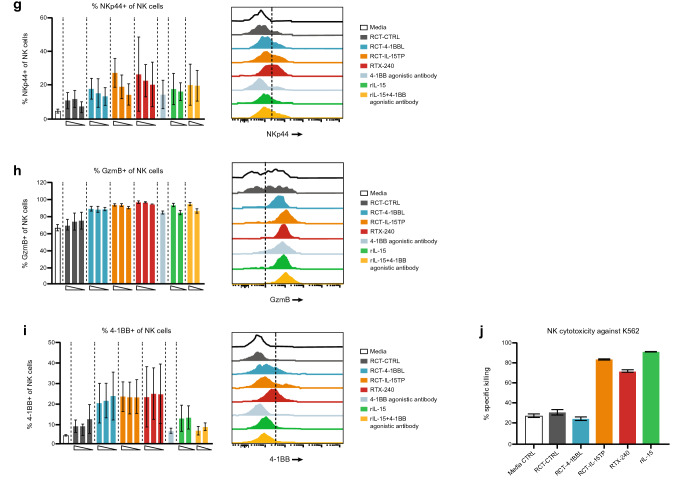
Direct activation of CD8 + T cells and NK cells in vitro. (**a**i) PBMCs were labeled with CTFR and incubated with the indicated treatments for 8 days, then analyzed by flow cytometry for: **a** memory CD8 + T cell numbers; **b** memory CD8 + T cell proliferation (percentage of cells that went through at least one division); **c** CD8 + effector memory differentiation; **d** NK cell numbers; **e** NK cell proliferation (percentage of cells that went through at least one division); **f**–**i** expression of the following molecules on NK cells: **f** TRAIL, **g** NKp44, **h** Granzyme B and **i** 4-1BB. **j** Purified NK cells were incubated in vitro overnight with the indicated treatments, then incubated with labeled K562 target cells for 4 h. Target cell killing was measured by flow cytometry. Bars indicate **a**–**i** SD of 3 biological replicates **j** or SD of 3 technical replicates. Flow plots are representative data from one donor. CTFR, CellTrace Far Red dye; GzmB, Granzyme B; IL-15TP, trans-presented interleukin-15; NK, natural killer; PBMC, peripheral blood mononuclear cell; rIL-15, recombinant IL-15; SD, standard deviation

Both soluble and membrane-bound IL-15 or 4-1BB agonists directly promote proliferation and cytotoxicity of CD8 + T cells [9, 24, 25] and NK cells [12, 26], showing increased activity when combined [9, 12]. Consistent with this, RTX-240 treatment of PBMCs in the absence of additional stimulation resulted in a fourfold increase in the number of memory CD8 + T cells (Fig. 3a), a tenfold increase in proliferation of CD45RO + memory CD8 + T cells (Fig. 3b) and a threefold increase in the percentage of effector memory CD8 + T cells (Fig. 3c) compared with RCT-CTRL. Similar effects were observed with 4-1BB agonist and rIL-15 but not with RCT-4-1BBL and RCT-IL-15TP.

PBMCs treated with RTX-240 showed dramatic increases in NK cell numbers (16-fold, Fig. 3d) and proliferation (eightfold, Fig. 3e) as compared with RCT-CTRL. These effects were present to a lesser extent with RCT-4-1BBL, RCT-IL-15TP, or positive controls.

NK cells can be triggered to kill target cells through engagement of death receptor ligands such as TRAIL, or natural cytotoxicity receptors such as NKp44 [27]. RTX-240 led to fivefold and twofold increases in NK cells expressing TRAIL and NKp44, respectively, compared with RCT-CTRL (Fig. 3f and g). Increased expression of the cytotoxic protease Granzyme B on NK cells was also demonstrated by treatment with RTX-240 (Fig. 3h). Similar effects were observed in NK cells after treatment with RCT-4-1BBL, RCT-IL-15TP, and positive controls. The percentage of 4-1BB + NK cells increased after treatment with RTX-240, RCT-4-1BBL and RCT-IL-15TP but not 4-1BB agonistic antibody or rIL-15 (Fig. 3i).

To evaluate the effect of RTX-240 on NK cell cytotoxicity, NK cells from healthy donors were co-cultured with RTX-240 or control treatments overnight, then incubated with K562 target cells. NK cells treated with RCT-IL-15TP or RTX-240 demonstrated 2.6–threefold increased killing of K562 targets versus media alone; this was also observed following rIL-15 treatment (Fig. 3j), as has been previously described [11].

Collectively, these data indicate that RTX-240 can directly stimulate CD8 + T cells and NK cells in vitro, leading to increased proliferation, activation and function, similar to the effects observed for treatment with 4-1BB agonist plus rIL-15. Furthermore, RTX-240 is superior to RCT-4-1BBL and RCT-IL-15TP, demonstrating additive or synergistic effects for the combination of ligands. The most pronounced effect of treatment with RTX-240 is the dramatic enhancement of NK cell expansion, while increasing NK cell functionality.

### *Effects of mRBC-240: an *in vivo* surrogate for RTX-240*

Owing to the rapid clearance of human RBCs in immunocompetent rodents [28, 29], RTX-240 cannot be effectively assessed in an animal model in vivo. Therefore, a murine surrogate for RTX-240 was developed to assess activity in an animal model in vivo. mRBC-240 comprises murine RBCs chemically conjugated with murine 4-1BBL and human IL-15TP. Surface expression of each molecule and the potential of mRBC-240 to activate 4-1BB and IL-15 downstream signaling was confirmed using binding and cell reporter assays in vitro. mRBC-240 demonstrated additive effects in expansion of CD8 + T cells and NK cells (supplementary Fig. 2). In addition, splenocytes stimulated with mRBC-240 in the presence of anti-CD3 produced higher levels of IFNγ compared with mRBC-IL-15TP and mRBC-4-1BBL (supplementary Fig. 2). Together, mRBC-240 displayed the bioactivity of RTX-240 for use as a surrogate in mouse studies.

#### *mRBC-240 distributes to the spleen and expands CD8* + *T cells and NK cells *in vivo

**Fig. 4:**
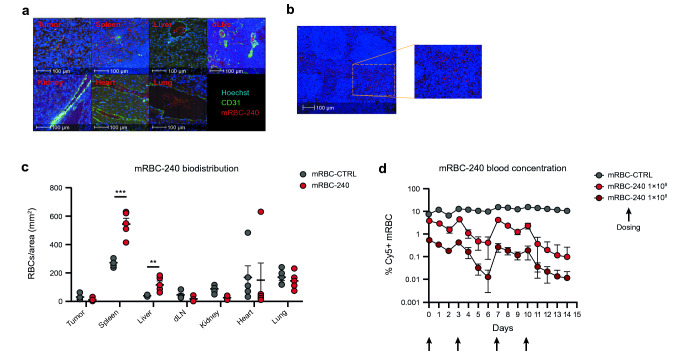

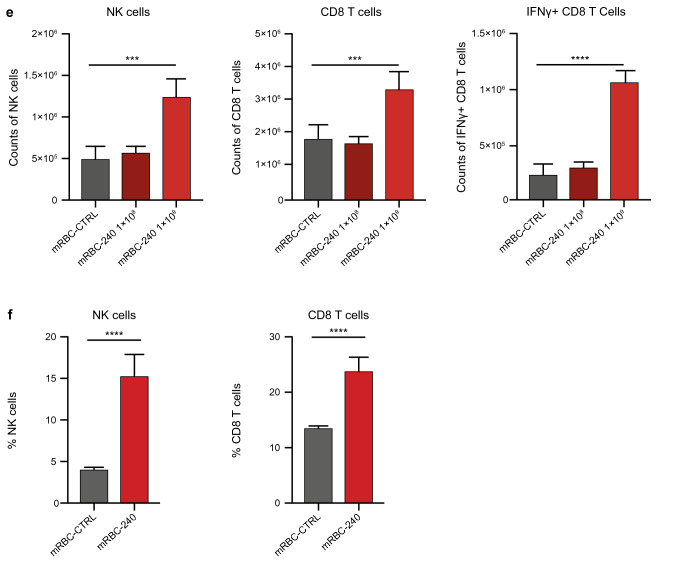
mRBC-240 biodistribution, PK and PD. **a** Representative images of mRBC-240 in different tissues (tumor, spleen, liver, draining lymph nodes, lung, kidney, and heart). mRBC-240 cells were labeled with CTFR prior to injection (red channel). Blood vessels were identified by CD31 staining (green channel) and nucleated cells were detected using Hoechst staining (blue channel). **b** Representative images of mRBC-240 in the red pulp of the spleen. **c** mRBC-240 biodistribution: quantification of the density (cells per mm^2^) of mRBC-240 and mRBC-CTRL in different organs 24 h following a single IV dose (*n* = 5). Data presented ± SEM **p < 0.01, ***p < 0.001, comparing mRBC-240 versus mRBC-CTRL. All comparisons were analyzed by t test. **d** PK of mRBC-240: concentration of mRBC-240 in the blood was assessed. Blood was collected 4 h post the first dose and each day thereafter and the percentage of Cy5-labeled mRBC-CTRL (1 × 10^9^) or mRBC-240 (1 × 10^9^ or 1 × 10^8^) in circulation was assessed by flow cytometry (*n* = 4 mice per group). **e** PD of mRBC-240 in the spleen of non-tumor-bearing mice: counts of total NK cells, CD8 + T cells and IFNγ + CD8 + T cells were analyzed by flow cytometry in the spleen on day 14 (*n* = 4 mice per group). **f** PD of mRBC-240 in the spleen of tumor-bearing mice (B16-F10): frequencies of NK cells and CD8 + T cells were analyzed by flow cytometry in the spleen on day 12 (*n* = 5 mice per group). Bars indicate SD of biological replicates. Comparisons were analyzed by a one-way ANOVA or unpaired T test and compared with mRBC-CTRL group and showing as **p < 0.01, ***p < 0.001, ****p < 0.0001. CTFR, CellTrace Far Red dye; IFNγ, interferon-γ; IV, intravenous; mRBC, mouse red blood cell; NK, natural killer; PD, pharmacodynamics; PK, pharmacokinetics; SD, standard deviation; SEM, standard error of the mean

In a biodistribution study of mRBC-240, after IV administration in mice, both mRBC-240 and mRBC-CTRL distributed predominantly to the spleen and specifically to the red pulp (Fig. 4a and b). The splenic red pulp capillaries feed an open circulation in which RBCs and other immune cells interact [30]. Importantly, the density of mRBC-240 in the spleen was higher than mRBC-CTRL, suggesting enhanced interaction of mRBC-240 with target cells in the spleen compared with mRBC-CTRL (Fig. 4c; *p* = 0.0002).

In most organs, mRBC-240 cells were found inside the vessels (Fig. 4c). Although mRBC-CTRL and mRBC-240 were detectable in the heart and lungs, these were localized to the lumen of the blood vessels, consistent with the absence of a perfusion step prior to tissue collection. In the liver, there was a small but statistically significant increase in mRBC-240 compared with mRBC-CTRL (*p* = 0.0063). mRBC-240 cells were detected at low levels in tumors, draining lymph nodes, and kidneys.

In a PK/PD evaluation for 14 days, while the average concentration of mRBC-CTRL in the blood was 12%, mRBC-240 cells were cleared faster with an average concentration of 1.7% for the 1 × 10^9^ dose and 0.17% for the 1 × 10^8^ dose (Fig. 4d).

We believe that faster clearance of mRBC-240 from the blood compartment is due to expression of 4-1BBL and IL-15TP on the surface of mRBC-240, which leads to interactions with immune cells in the blood and spleen. In the blood, to determine mRBC-240 PK, we use a flow cytometry assay that is based on RBC singlets. Thus, when mRBC-240 cells interact with immune cells in the blood, mRBC-240 cells are not detected by the PK assay, which may be a contributor to the short PK of mRBC-240 compared with mRBC-CTRL. In addition, expression of 4-1BBL and IL-15TP on mRBC-240 leads to interaction of mRBC-240 with immune cells predominantly in the spleen and sequestration of mRBC-240 there, as suggested by the biodistribution study (Fig. 4c). In non-tumor-bearing mice mRBC-240 induced a dose-dependent, 2–2.5 fold increase in expansion of splenic NK cells and CD8 + T cells compared with mRBC-CTRL (*p* = 0.0002 and *p* = 0.0009, respectively). Furthermore, mRBC-240 treatment induced a fivefold increase in count of IFNγ-producing CD8 + T cells (*p* = 0.0001) (Fig. 4e). This suggests that mRBC-240 can recapitulate, in vivo, the effects seen with RTX-240 and mRBC-240 in vitro. Increased frequency of NK cells and CD8 + T cells in the spleen was similarly observed in tumor-bearing mice (*p* < 0.0001) (Fig. 4f), indicating that despite the observation that mRBC-240 cells do not distribute to the tumor microenvironment, mRBC-240 can activate CD8 + T cells and NK cells in the spleen, which may lead to tumor growth inhibition.

#### *mRBC-240 promotes tumor control *in vivo

**Fig. 5:**
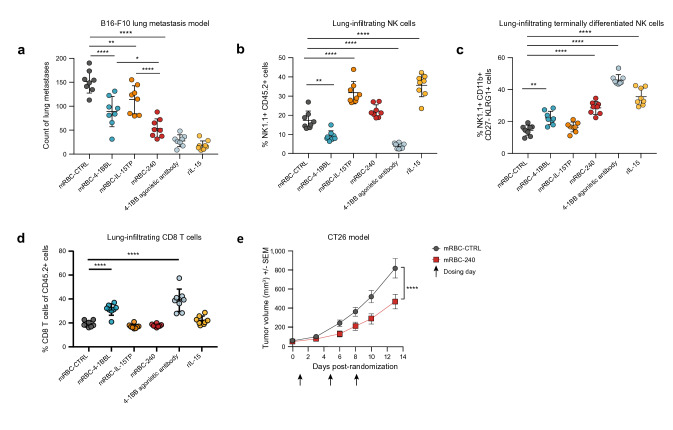

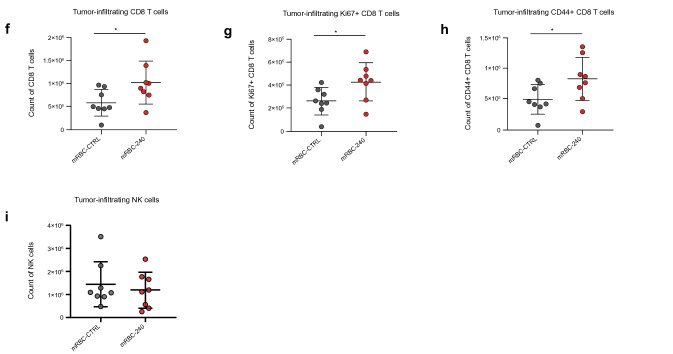
mRBC-240 promotes tumor control in B16-F10 and CT26 models. **a** Lung metastases enumeration in C57BL/6 mice inoculated IV with B16-F10 tumor cells. Analysis performed on day 14 (*n* = 8 mice/group). The frequency of **b** NK cells (NK1.1 +) and **c** terminally differentiated NK cells (NK1.1 + CD11b + CD27-KLRG1 +) and **d** CD8 + T cells in the lung were analyzed by flow cytometry. Data presented ± SD and comparisons analyzed by one-way ANOVA *p < 0.05, **p < 0.01, ***p < 0.001, ****p < 0.0001. **e** Tumor growth curves for CT26 tumor model in BALB/c mice. Data presented ± SEM ****p < 0.0001, comparisons were analyzed by two-way ANOVA. The numbers of **f** tumor-infiltrating CD8 + T cells, **g** proliferating (Ki67 +) CD8 + T cells and **h** activated (CD44 +) CD8 + T cells as well as **i** tumor-infiltrating NK cells were analyzed by flow cytometry on day 11 (*n* = 8 mice per group). Data presented ± SD *p < 0.05. Comparisons were analyzed by unpaired T test. IL-15TP, trans-presented interleukin 15; IV, intravenous; mRBC, mouse red blood cell; NK, natural killer; rIL-15, recombinant IL-15; SD, standard deviation; SEM, standard error of the mean

mRBC-240 anti-tumor activity was evaluated in vivo in two tumor models in mice. In the B16-F10 melanoma lung metastasis model [31], 3 doses of 1 × 10^9^ mRBC-240 (days 1, 5 and 8 post cancer-cell inoculation) significantly reduced tumor burden compared with mRBC-CTRL (*p* < 0.0001). mRBC-240 was more effective in reducing lung metastases than mRBC-4-1BBL (*p* = 0.0179) or mRBC-IL-15TP (*p* < 0.0001), demonstrating an advantage for co-presentation of 4-1BBL and IL-15TP on mRBC-240 (Fig. 5a). The positive controls rIL-15 and 4-1BB agonistic antibody showed fewer metastases compared with mRBC-CTRL and mRBC-240.

There was a significant increase in the percentage of lung-infiltrating NK cells on day 14 in mice treated with mRBC-IL-15TP (*p* < 0.0001) (Fig. 5b). A similar, not statistically significant, trend was observed in mice treated with mRBC-240. Finally, mRBC-240 treatment led to a significant increase in the percentage of terminally differentiated NK cells in the lungs (NK1.1 + CD11b + CD27-KLRG1 + ; *p* < 0.0001), which was also observed in mice treated with mRBC-4-1BBL (*p* = 0.0016) but not mRBC-IL-15TP (Fig. 5c), suggesting an additive effect of 4-1BBL and IL-15TP on mRBC-240. No change in lung infiltration of CD8 + T cells was observed in mRBC-240 treatment compared to mRBC-CTRL (Fig. 5d).

In the CT26 model of colon cancer, three doses of mRBC-240 inhibited tumor growth in mice compared with mRBC-CTRL (*p* < 0.0001), with a reduced tumor burden at day 13 (Fig. 5e). Immune cell profiling on day 11 showed that mRBC-240 significantly expanded CD8 + T cells in the tumor compared with mRBC-CTRL (*p* = 0.038) (Fig. 5f). Tumor-infiltrating CD8 + T cells showed higher proliferation (Ki67 +) and enhanced activation (CD44 +) in mRBC-240 treated mice compared with mRBC-CTRL (*p* = 0.044 and *p* = 0.034, respectively) (Fig. 5g and h). However, no change in tumor infiltration of NK cells was observed in mRBC-240 treatment compared to mRBC-CTRL (Fig. 5i).

#### *mRBC-240 is well-tolerated *in vivo

In clinical studies, 4-1BB agonists induce multiple toxicities [32], including liver inflammation. In mice lymphopenia, increased monocyte count, thrombocytopenia and anemia were also reported [33]. ALT-803, a fusion protein of IL-15R and IL-15, was reported to be tolerated with no dose-limiting toxicities in a phase I study [34], although toxicities in mice included weight loss, increased lymphocyte count, and spleen hyperplasia [10]. Body and organ weights from B16-F10 tumor-bearing mice treated with mRBC-240 were unchanged compared with mRBC-CTRL (supplementary Fig. 3). In contrast, rIL-15 and 4-1BB agonistic antibody treatments led to an increase in spleen weights (IL-15: *p* = 0.0001, 4-1BB agonist: *p* = 0.0048). Additionally, rIL-15 treatment led to lower body weight (*p* = 0.0096) and increased lung weight (*p* = 0.02) compared with mRBC-CTRL-treated mice. These data suggest that mRBC-240 is better tolerated than 4-1BB agonistic antibody or rIL-15.

**Fig. 6:**
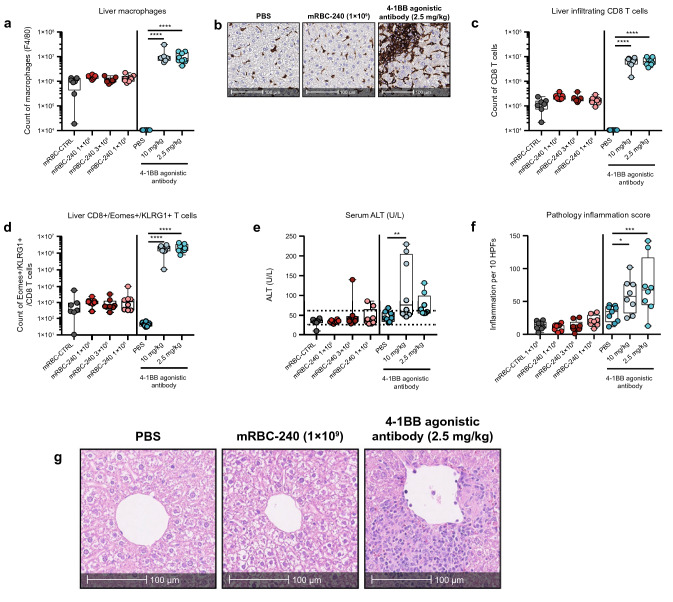
mRBC-240 is tolerated in mice. **a** Quantification of liver macrophages (F4/80 +). **b** Representative images of IHC staining of macrophages (F4/80) from selected mice. **c** Quantification of liver-infiltrating CD8 + T cells and **d** liver-infiltrating cytotoxic CD8 + T cells (CD8 + /Eomes + /KLRG1 +). Quantification was performed on liver samples of C57BL/6 wild-type mice by flow cytometry on day 18 following 4 doses of either mRBC-240 (1 × 10^9^, 3 × 10^8^, or 1 × 10^8^), mRBC-CTRL, 4-1BB agonistic antibody (10 mg/kg or 2.5 mg/kg) (*n* = 8 mice/group) or PBS. **e** ALT liver enzyme levels (U/L) in serum on day 18. **f** Inflammation scoring performed on H&E stained liver sections. **g** Representative images of H&E staining of liver sections from selected mice. All comparisons were analyzed by a one-way ANOVA and compared with control groups and showing as *p < 0.05, **p < 0.01, ***p < 0.001, ****p < 0.0001. ALT, alanine aminotransferase; IHC, immunohistochemistry; H&E, hematoxylin and eosin; mRBC, mouse red blood cell; PBS, phosphate-buffered saline

A previously described liver toxicity model that assessed a 4-1BB agonistic antibody [17] was adapted to evaluate toxicity of mRBC-240. In this non-good laboratory practice (GLP) safety study comparing toxicity induced by mRBC-240 or a 4-1BB agonistic antibody, mRBC-240 was determined to be tolerated compared with the 4-1BB agonistic antibody. No significant changes in macrophage infiltration into the liver of mRBC-240-treated mice were observed on day 18 of the study compared with negative controls. A significant increase in macrophage infiltration was seen for the 4-1BB agonistic antibody-treated animals at both dose levels compared with PBS-treated control (*p* = 0.0001) (Fig. 6a; IHC: Fig. 6b).

No significant changes in liver-infiltrating CD8 + T cells or liver CD8 + /Eomes + /KLRG1 + T cells – drivers of liver injury – were observed in this study in mice that received four doses of mRBC-240 compared with controls (Fig. 6c and d). In contrast, 4-1BB agonistic antibody-treated animals at both dose levels showed a statistically significant increase in CD8 + T cell infiltration into the liver compared with control (*p* = 0.0001) and a significant increase in liver-infiltrating CD8 + /Eomes + /KLRG1 + T cells (*p* = 0.0001).

Mice treated with mRBC-240 in this study showed ALT within the normal range in 21/24 animals (87.5% of mice, no significant difference from control). 4-1BB agonistic antibody-treated mice showed elevated ALT levels in 9/16 mice (56%, *p* = 0.0017 for 4-1BB agonistic antibody at 10 mg/kg; Fig. 6e). 4-1BB agonistic antibody-treated groups showed a statistically significant increase in inflammation score (*p* = 0.035 and *p* = 0.0004 for 2.5 mg/kg and 10 mg/kg, respectively) (Fig. 6f). This change in the liver inflammation score was not observed in groups treated with mRBC-240. H&E staining of liver sections demonstrated perivascular inflammation in liver sections from 4-1BB agonistic antibody-treated animals (Fig. 6g); similar peri-vascular inflammation was not observed in mRBC-240-treated mice. Complete blood count (CBC) revealed no changes in white blood cell, lymphocyte or monocyte counts, or hemoglobin levels in mice that received mRBC-240 compared with mRBC-CTRL (supplementary Fig. 4). 4-1BB agonistic antibody treatment significantly decreased white blood cell and lymphocyte counts and increased monocytes when compared with PBS-treated mice (supplementary Fig. 4).

## Discussion

The development of RTX-240 was based on three concepts: that normal physiological distribution of RBCs would avoid unwanted off-tissue effects and lower the potential for toxicity; that a combination of multiple ligands would provide improved activity; and that expression of ligands in their natural, cell-surface conformation would facilitate binding to receptors through cell-to-cell contact and lead to more potent activation of downstream signaling pathways. The data presented here demonstrate that 4-1BBL and IL-15TP co-expressed on the cell surface of RTX-240 activated both 4-1BB and IL-15 receptors. Dual expression did not compromise signaling through either receptor pathway and acted synergistically to activate NK cells and T cells in vitro. A murine surrogate of RTX-240 recapitulated these activities in vitro and in vivo and demonstrated superior efficacy and tolerability compared with exogenous ligand treatment in multiple in vivo models.

A key property of engineered RBCs for clinical application is their biodistribution—they follow the same distribution and clearance patterns as endogenous RBCs, with minimal organ accumulation [35]. RBCs, lymphocytes and all other circulating cells freely percolate through the red pulp and are subsequently re-collected into the sinuses for venous drainage [36]. In line with these findings, biodistribution assays indicated that mRBC-240 was found in the circulation and the spleen. The low flow rate and known mixing of lymphocytes and RBCs within the red pulp, combined with the observed increase in mRBC-240 at this location, suggest that the spleen red pulp is the primary area of mRBC-240 interaction with T and NK cells. This limited biodistribution is likely to reduce the unwanted off-tumor effects of RTX-240. Despite the absence of mRBC-240 in tumors, expansion and activation of NK cells and CD8 + T cells was observed in both the spleen and the tumor in mRBC-240-treated mice, suggesting that these immune cells are activated by mRBC-240 in the spleen and then traffic to the tumor. This is in line with recent reports indicating that T cells activated in the periphery can traffic to the tumor and potentially drive anti-tumor responses [37–40].

Preclinical studies with the IL-15 super-agonist ALT-803 (now N-803) demonstrated the ability of IL-15–IL-15Rα complexes to expand and activate human NK cells and T cells [10] and to enhance NK-mediated cytotoxicity toward K562 targets in vitro [11]. Clinical studies with ALT-803 also demonstrated in vivo expansion of CD8 + T cells and NK cells [34, 41, 42]. Similarly, 4-1BB activation enhances proliferation of CD8 + T cells and anti-tumor activity [43]. However, although ALT-803 is well-tolerated by patients [41], some 4-1BB agonistic antibodies have shown dose-dependent liver toxicity that limited the clinical development of these therapies [32, 44]. Toxicity was seen with both exogenous ligands in our studies, but was not observed with membrane-bound ligands presented on mRBC-240, which showed efficacy in tumor models as well as a favorable therapeutic index. Crucially, mRBC-240 cells were tolerated by mice and showed no liver or hematological toxicities, no increase in organ weights, no increase in liver infiltration of CD8 + T cells or macrophages, no change in liver inflammation score and no change in CBC. In contrast, the 4-1BB agonistic antibody resulted in increased liver infiltration of CD8 + T cells and macrophages, increased serum transaminases, increased liver inflammation score and peri-vascular inflammation and lymphopenia, all of which are consistent with previous reports [10, 17, 32, 33, 44]. Favorable tolerability of mRBC-240 compared with the 4-1BB agonistic antibody may be explained by the cells’ biodistribution: mRBC-240 did not activate liver Kupffer cells, which secrete IL-27 and are required for the initiation of the toxic cascade [17]. Collectively, these data support the hypothesis that RTX-240 may be a better tolerated therapy than a 4-1BB agonistic antibody or rIL-15.

Multiple lines of evidence support the combination of IL-15 and 4-1BBL for improved potency and efficacy. The independent activity of both IL-15 fusions [45, 46] and 4-1BB agonists [47–50] has been demonstrated for in vivo tumor inhibition; however, there is a clear benefit for the simultaneous presentation of IL-15 and 4-1BBL in a single fusion protein in a tumor mouse model in vivo [9]. Here, we demonstrate that the combination of 4-1BBL and IL-15TP on mRBC-240 inhibited tumor growth in the B16-F10 model of lung metastasis and this was accompanied by an increase in the frequency of NK cells and terminally differentiated NK cells in the lung, as has been observed for rIL-15. mRBC-240 was more efficacious than cells presenting only 4-1BBL or IL-15TP, emphasizing the benefit of having both molecules expressed on the same cell and in line with former demonstrations [9]. In a second tumor model (CT26 colon cancer), mRBC-240 showed tumor growth inhibition that was associated with an increase in activated and proliferating CD8 + T cells in the tumor. These separate in vivo observations support the combination of 4-1BBL and IL-15TP for improved efficacy and suggest that the RTX-240 mechanism of action includes expansion of NK cells and CD8 + T cells in the spleen and increased infiltration of activated NK cells and CD8 + T cells in the tumor site.

Clinical studies have linked increased activation and cytotoxic function of NK cells with improved patient prognosis in several cancer types [51–53]. In vitro studies with human PBMCs support the use of a combination of IL-15 and 4-1BBL for robust activation of NK and CD8 + T cells. In the presence of TCR stimulation, RTX-240 increased CD8 + T cell proliferation and activation primarily through 4-1BBL. In the absence of TCR stimulation, both 4-1BBL and IL-15TP were required for greatest memory CD8 + T cell proliferation, specifically effector memory CD8 + T cells and greatest NK cell activation, proliferation and expression of cytotoxic molecules. In a short-term cytotoxicity assay, RTX-240 increased NK target cell killing exclusively through IL-15TP. In this experiment, RTX-240 primed NK cells to be more cytotoxic against the myeloid leukemia line, K562, demonstrating the potential activity of RTX-240 in activating NK cells against leukemic cells.

Feeder cells consisting of K562 expressing 4-1BBL and membrane-bound IL-15 have been used to expand NK cells for adoptive cell therapy for cancer, inducing upregulation of TRAIL, NKp44 and 4-1BB [12, 26]. RTX-240 similarly induced dramatic expansion of NK cells and expression of these activating receptors on NK cells in vitro, suggesting that RTX-240 may be able to leverage the synergistic effects of 4-1BBL and IL-15TP to drive NK cell expansion and activation in patients.

Cell-surface presentation of 4-1BBL and IL-15 may further enhance their combined activity, compared with soluble agonists. Notably, RTX-240 treatment led to greater NK cell and CD8 + T cell expansion in vitro than 4-1BB agonistic antibody, rIL-15, or a combination of both, suggesting that natural ligand presentation of 4-1BBL and IL-15TP on RTX-240 may be more stimulatory.

In summary, this study highlights the potential of RTX-240 as a novel anticancer cell therapy to enable 4-1BB- and IL-15-driven efficacy. The data demonstrate the many advantages of RTX-240, including a specific biodistribution to the spleen red pulp that may limit off-tissue toxicity and the ability to mediate cell-to-cell interactions and to present ligands and cytokines in their native conformation for more potent activation of the immune system. The in vitro and in vivo results presented here offer promise for clinical translation and warrant evaluation of RTX-240 in patients with cancer. Based on these data, a clinical trial has been initiated (NCT04372706).

## Supplementary Information

Below is the link to the electronic supplementary material.

Supplementary file1 (PDF 1083 kb)

## Abbreviations

ALT: Alanine aminotransferase
CBC: Complete blood count
CTFR: CellTrace Far Red dye
GLP: Good laboratory practice
GPA: Glycophorin A
H&E: Hematoxylin and eosin
IFNγ: Interferon-γ
IL-15: Interleukin 15
IL-15Rα: IL-15 receptor α
IL-15TP: Trans-presented IL-15
IP: Intraperitoneal
IV: Intravenous
NK: Natural killer
PBMC: Peripheral blood mononuclear cell
PBS: Phosphate-buffered saline
PK: Pharmacokinetics
RBC: Red blood cell
rIL-15: Recombinant IL-15

## References

[1] Darvin P, Toor SM, Nair VS, Elkord E (2018). Immune checkpoint inhibitors: recent progress and potential biomarkers. Exp Mol Med.

[2] Chavez JC, Bachmeier C, Kharfan-Dabaja MA (2019). CAR T-cell therapy for B-cell lymphomas: clinical trial results of available products. Ther Adv Hematol.

[3] Alexander W (2016). The Checkpoint immunotherapy revolution. Pharm Ther.

[4] Bonifant CL, Jackson HJ, Brentjens RJ, Curran KJ (2016). Toxicity and management in CAR T-cell therapy. Mol Ther.

[5] Li Y, Sun R (2018) Tumor immunotherapy: New aspects of natural killer cells. Chin J Cancer Res 30:173–196. 10.21147/j.issn.1000-9604.2018.02.02 10.21147/j.issn.1000-9604.2018.02.02 PMC5953955 29861604

[6] Pulle G, Vidric M, Watts TH (2006). IL-15-dependent Induction of 4–1BB Promotes Antigen-Independent CD8 Memory T Cell Survival. J Immunol.

[7] Vidard L, Dureuil C, Baudhuin J (2019). CD137 (4–1BB) Engagement Fine-Tunes Synergistic IL-15– and IL-21–Driven NK Cell Proliferation. J Immunol.

[8] Curtsinger JM, Mescher MF (2010). Inflammatory cytokines as a third signal for T cell activation. Curr Opin Immunol.

[9] Kermer V, Hornig N, Harder M (2014). Combining antibody-directed presentation of IL-15 and 4–1BBL in a trifunctional fusion protein for cancer immunotherapy. Mol Cancer Ther.

[10] Rhode PR, Egan JO, Xu W (2016). Comparison of the Superagonist Complex, ALT-803, to IL15 as Cancer Immunotherapeutics in Animal Models. Cancer Immunol Res.

[11] Rosario M, Liu B, Kong L (2016). The IL-15-based ALT-803 complex enhances fcγRIIIa-triggered NK cell responses and in vivo clearance of B cell lymphomas. Clin Cancer Res.

[12] Fujisaka H, Kakuda H, Shimasaki N (2009). Expansion of highly cytotoxic human natural killer cells for cancer cell therapy. Cancer Res.

[13] Lapteva N, Durett AG, Sun J (2012). Large-scale ex vivo expansion and characterization of natural killer cells for clinical applications. Cryotherapy.

[14] Segal NH, He AR, Doi T (2018). Phase I study of single-agent utomilumab (PF-05082566), a 4-1BB/CD137 agonist, in patients with advanced cancer. Clin Cancer Res.

[15] Tolcher AW, Sznol M, Hu-Lieskovan S (2017). Phase Ib study of utomilumab (PF-05082566), a 4–1BB/CD137 agonist, in combination with pembrolizumab (MK-3475) in patients with advanced solid tumors. Clin Cancer Res.

[16] Bartkowiak T, Curran MA (2015). 4–1BB agonists: multi-potent potentiators of tumor immunity. Front Oncol.

[17] Bartkowiak T, Jaiswal AR, Ager CR (2018). Activation of 4–1BB on liver myeloid cells triggers hepatitis via an interleukin-27–dependent pathway. Clin Cancer Res.

[18] Imai C, Iwamoto S, Campana D (2005). Genetic modification of primary natural killer cells overcomes inhibitory signals and induces specific killing of leukemic cells. Blood.

[19] Huang N-J, Pishesha N, Mukherjee J (2017). Genetically engineered red cells expressing single domain camelid antibodies confer long-term protection against botulinum neurotoxin. Nat Comms.

[20] Shi J, Kundrat L, Pishesha N (2014). Engineered red blood cells as carriers for systemic delivery of a wide array of functional probes. PNAS.

[21] Olbrich H, Slabik C, Stripecke R (2017). Reconstructing the immune system with lentiviral vectors. Virus Genes.

[22] Milone MC, O’Doherty U (2018). Clinical use of lentiviral vectors. Leukemia.

[23] Kolb HC, Finn MG, Sharpless KB (2001). Click chemistry: diverse chemical function from a few good reactions. Angew Chemie.

[24] Steel JC, Waldmann TA, Morris JC (2011). Interleukin-15 biology and its therapeutic implications in cancer. Trends Pharmacol Sci.

[25] Croft M (2009). The role of TNF superfamily members in T-cell function and diseases. Nat Rev Immunol.

[26] Zhang H, Cui Y, Voong N (2011). Activating signals dominate inhibitory signals in CD137L/IL-15 activated natural killer cells. J Immunother.

[27] Zamai L, Ponti C, Mirandola P (2007). NK Cells and Cancer J Immunol.

[28] Straat M, Klei T, de Korte D (2015). Accelerated clearance of human red blood cells in a rat transfusion model. Intensive Care Med Exp.

[29] Hu Z, Van Rooijen N, Yang Y-G (2011). Macrophages prevent human red blood cell reconstitution in immunodeficient mice. Blood.

[30] Steiniger B, Bette M, Schwarzbach H (2011). The open microcirculation in human spleens. J Histochem Cytochem.

[31] Overwijk WW, Refisto NP (2001). B16 as a mouse model for human melanoma. Curr Protoc Immunol.

[32] Chester C, Sanmamed MF, Wang J, Melero I (2018). Immunotherapy targeting 4–1BB: mechanistic rationale, clinical results and future strategies. Blood.

[33] Niu L, Strahotin S, Hewes B (2007). Cytokine-mediated disruption of lymphocyte trafficking, hemopoiesis and induction of lymphopenia, anemia and thrombocytopenia in anti-CD137-treated mice. J Immunol.

[34] Romee R, Cooley S, Berrien-Elliott MM (2018). First-in-human phase 1 clinical study of the IL-15 superagonist complex ALT-803 to treat relapse after transplantation. Blood.

[35] Chapanian R, Constantinescu I, Brooks DE (2012). In vivo circulation, clearance and biodistribution of polyglycerol grafted functional red blood cells. Biomater.

[36] Steiniger BS (2015). Human spleen microanatomy: why mice do not suffice. Immunol.

[37] Yost KE, Satpathy AT, Wells DK (2019). Clonal replacement of tumor-specific T cells following PD-1 blockade. Nat Med.

[38] Wu TD, Madireddi S, de Almeida PE (2020). Peripheral T cell expansion predicts tumour infiltration and clinical response. Nature.

[39] Pauken KE, Shahid O, Lagattuta KA (2021). Single-cell analyses identify circulating anti-tumor CD8 T cells and markers for their enrichment. J Exp Med.

[40] Beltra JC, Manne S, Abdel-Hakeem MS (2020). Developmental relationships of four exhausted CD8+ T cell subsets reveals underlying transcriptional and epigenetic landscape control mechanisms. Immun.

[41] Margolin K, Morishima C, Velcheti V (2018). Phase i trial of ALT-803, a novel recombinant IL15 complex, in patients with advanced solid tumors. Clin Cancer Res.

[42] Wrangle JM, Velcheti V, Patel MR (2018). ALT-803, an IL-15 superagonist, in combination with nivolumab in patients with metastatic non-small cell lung cancer: a non-randomised, open-label, phase 1b trial. Lancet Oncol.

[43] Melero I, Shuford WW, Newby SA (1997). Monoclonal antibodies against the 4–1BB T-cell activation molecule eradicate established tumors. Nat Med.

[44] Segal NH, Logan TF, Hodi FS (2017). Results from an integrated safety analysis of urelumab, an agonist anti-CD137 monoclonal antibody. Clin Cancer Res.

[45] Dubois S, Patel HJ, Zhang M (2008). Preassociation of IL-15 with IL-15Rα-IgG1-Fc enhances its activity on proliferation of NK and CD8+/CD44high T cells and its antitumor action. J Immunol.

[46] Bessard A, Sole V, Bouchard G (2009). High antitumor activity of RLI, an interleukin-15 (IL-15)–IL-15 receptor α fusion protein, in metastatic melanoma and colorectal cancer. Mol Cancer Ther.

[47] Claus C, Ferrara C, Xu W, et al (2019) Tumor-targeted 4–1BB agonists for combination with T cell bispecific antibodies as off-the-shelf therapy. Sci Transl Med 11:eaav5989. 10.1126/scitranslmed.aav5989 10.1126/scitranslmed.aav5989 PMC7181714 31189721

[48] Houot R, Goldstein MJ, Kohrt HE (2009). Therapeutic effect of CD137 immunomodulation in lymphoma and its enhancement by treg depletion. Blood.

[49] Chu D-T, Bac ND, Nguyen K-H (2019). An update on anti-CD137 antibodies in immunotherapies for cancer. Int J Mol Sci.

[50] Gauttier V, Judor J-P, Le Guen V (2014). Agonistic anti-CD137 antibody treatment leads to antitumor response in mice with liver cancer. Int J Cancer.

[51] Liljefors M, Nilsson B, Skog A-L (2003). Natural killer (NK) cell function is a strong prognostic factor in colorectal carcinoma patients treated with the monoclonal antibody 17–1A. Int J Cancer.

[52] Beano A, Signorino E, Evangelista A (2008). Correlation between NK function and response to trastuzumab in metastatic breast cancer patients. J Transl Med.

[53] Pasero C, Gravis G, Granjeaud S, et al (2015) Highly effective NK cells are associated with good prognosis in patients with metastatic prostate cancer. Oncotarget 6:14360–14393. 10.18632/oncotarget.3965 10.18632/oncotarget.3965 PMC4546472 25961317

