# Supplementary material for: Anti-tumor effects of RTX-240: an engineered red blood cell expressing 4-1BB ligand and interleukin-15
Source: Cancer Immunol Immunother. 2021 Jul 9;70(9):2701–19. doi: 10.1007/s00262-021-03001-7 (PMC8360899; doi:10.1007/s00262-021-03001-7)
Supplement: Supplementary file 1 — Supplementary file1 (PDF 1083 kb) [file 262_2021_3001_MOESM1_ESM.pdf]

Anti-tumor effects of RTX-240: an engineered red blood cell expressing 4-1BB ligand and interleukin-15

Supplementary material

Supplementary table 1: Antibodies used for flow cytometry

**Anti-mouse antibodies**	**Clone**	**Source**
CD3	17A2	BioLegend
CD8	53-6.7	BD Biosciences / BioLegend
NK1.1	PK136	BioLegend
CD44	IM7	BioLegend
CD45.2	104	BioLegend
CD45	30-F11	BD Biosciences
Ki67	B56	BD Biosciences
KLRG1	2F1/KLRG1	BioLegend
EOMES	Dan11mag	Thermo Fisher Scientific
F4/80	EMR1	BioLegend
CD27	LG.3A10	BioLegend
CD11b	M1/70	BioLegend

**Anti-human antibodies**	**Clone**	**Source**
Anti-human CD3	UCHT1	BD Biosciences
Anti-human CD56	5.1H11	BioLegend

Anti-human CD45RO	UCHL1	BioLegend
Anti-human CD62L	DREG-56	BioLegend
Anti-human CD8	RPA-T8	BioLegend
Anti-human TRAIL	RIK-2	BioLegend
Anti-human CD56	5.1H11	BioLegend
Anti-human NKp44	44.189	Thermo Fisher Scientific
Anti-human 4-1BB	4B4-1	BioLegend
Anti-human IFNγ	4S.B3	BioLegend
Anti-human GZMB	GB11	BioLegend
Anti-mouse IL-15Rα	DNT15Ra	Thermo Fisher Scientific
Anti-human 4-1BBL	5F4	BioLegend

IFNγ, interferon γ; NK, natural killer

Supplementary table 2: Gating strategies for flow cytometry

Cell type	Gating strategy (murine)
All cells	
All immune cells	CD45.2+ of live cells (LD-negative population)
CD8	CD8+ of live CD45.2+
Proliferating CD8+ T cells	Ki67+ of CD8+ T cells
Activated CD8+ T cells	CD44+ or IFNγ of CD8+ T cells
Liver CD8+ T cells associated with liver injury	Eomes+/KLRG1+ of CD8+ T cells
Liver macrophages	F4/80+ of live CD45.2+
NK	NK1.1+ of live CD45.2+
Terminally differentiated NK	CD11b+/CD27-/KLRG1+ of NK1.1+

Cell type	Gating strategy (human)
Leukocytes	FSC/SSC gating
Live cells	LD-negative population
CD8	CD3+/CD8+ of live cells
Proliferating CD8+ T cells	CTFR diluted of CD3+/CD8+
Activated CD8	GZMB+ of CD3+/CD8+

CD8 memory T cells	CD45RO+/CD8+/CD3+
Proliferating memory CD8+ T cells	CTFR diluted of CD45RO+/CD8+/CD3+
TEM	CD45RO+/CD62L- of CD8+ T cells
NK	CD56+/CD3- of live cells
Proliferating NK cells	CTFR diluted of CD56+/CD3-
Activated NK	TRAIL+ or NKp44+ or 4-1BB+ or GZMB+ or IFNγ of NK

CTFR, CellTrace™ Far Red dye; IFNγ, interferon γ; NK, natural killer

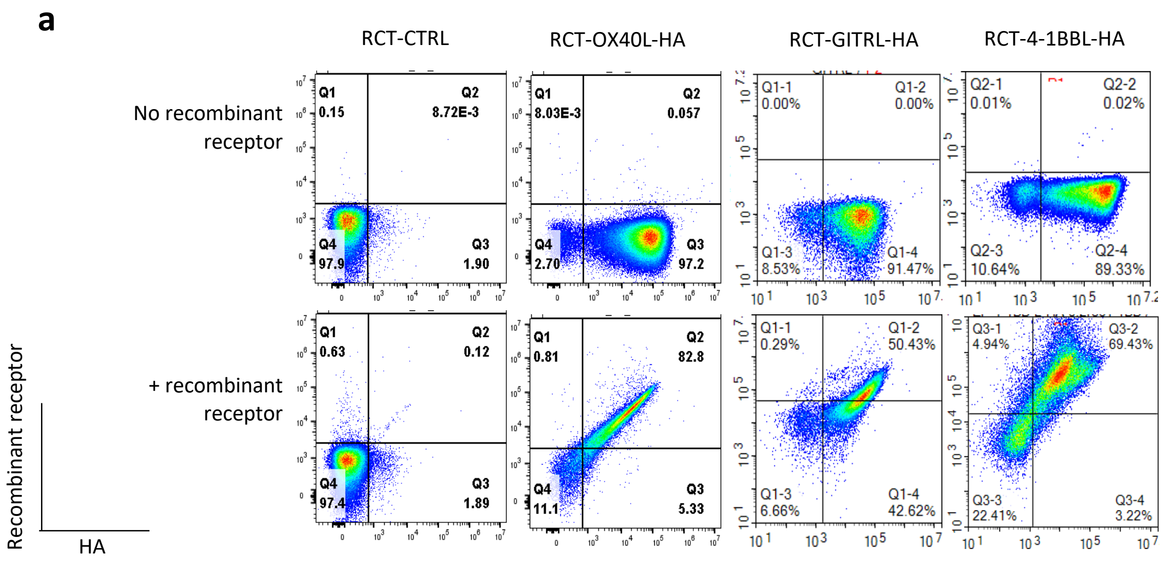
a
RCT-CTRL
RCT-OX40L-HA
RCT-GITRL-HA
RCT-4-1BBL-HA
No recombinant receptor
+ recombinant receptor
Recombinant receptor
HA
Q1 0.15
Q2 8.72E-3
Q4 97.9
Q3 1.90
Q1 8.03E-3
Q2 0.057
Q4 2.70
Q3 97.2
Q1-1 0.00%
Q1-2 0.00%
Q1-3 8.53%
Q1-4 91.47%
Q2-1 0.01%
Q2-2 0.02%
Q2-3 10.64%
Q2-4 89.33%
Q1 0.63
Q2 0.12
Q4 97.4
Q3 1.89
Q1 0.81
Q2 82.8
Q4 11.1
Q3 5.33
Q1-1 0.29%
Q1-2 50.43%
Q1-3 6.66%
Q1-4 42.62%
Q3-1 4.94%
Q3-2 69.43%
Q3-3 22.41%
Q3-4 3.22%

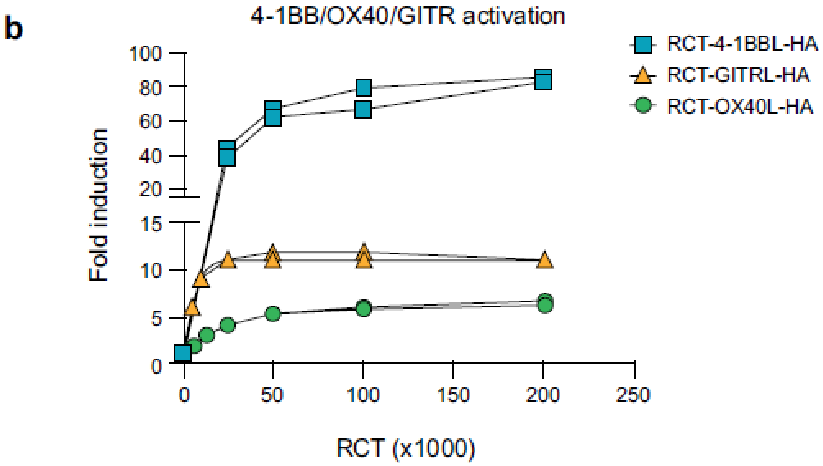
b
4-1BB/OX40/GITR activation
RCT-4-1BBL-HA
RCT-GITRL-HA
RCT-OX40L-HA
Fold induction
RCT (x1000)
0
5
10
15
20
40
60
80
100
50
150
200
250

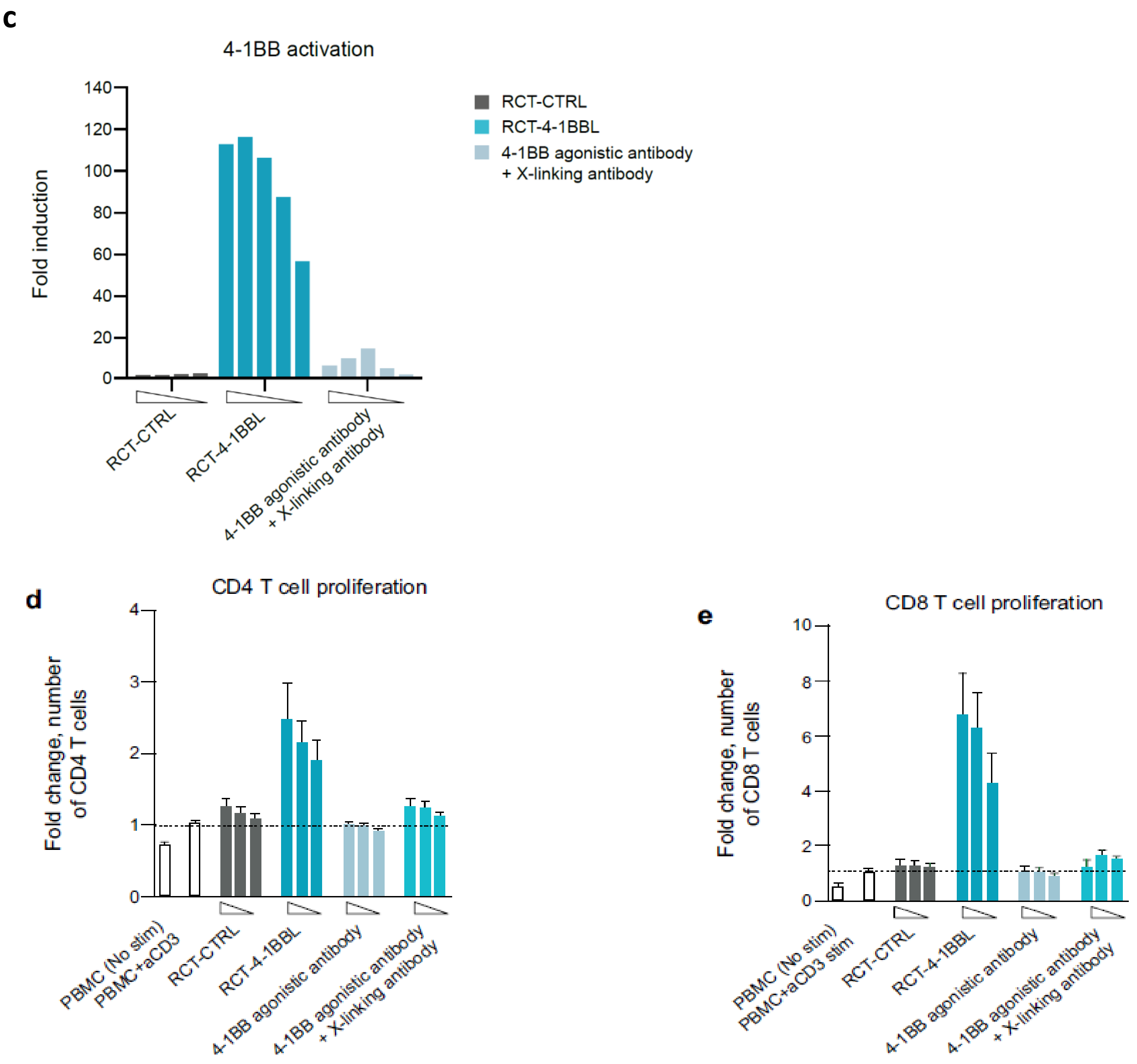

Supplementary figure 1: Engineered red blood cells expressing functional costimulatory ligands

a Representative flow cytometry plots for RCT-CTRL, RCT-OX40L-HA, RCT-GITRL-HA and RCT-4-1BBL-HA incubated in PBS with or without His-tagged recombinant receptors, stained with anti-HA antibody and anti-His antibody, and evaluated for expression of HA-tagged therapeutic proteins (top panel) and binding to recombinant His-tagged receptors (lower panel). **b** RCTs

expressing 4-1BBL, GITRL or OX40L were incubated with NFκB reporter Jurkat cells expressing 4-1BB, GITR or OX40, respectively, for 6 hours, and NFκB activation was measured. Bars indicate technical replicates. **c** NFκB activation in reporter Jurkat cells incubated for 6 hours with engineered RBCs (2-fold dilutions from 4 x 10^5 cells) or 4-1BB agonistic antibody or isotype control antibody (10-fold dilutions from 100 nM) with crosslinker (10-fold dilutions from 250 nM). **d, e** 2 x 10^5 PBMCs were cultured for 3 days with 0.5 µg/mL anti-CD3 and engineered RBCs (2-fold dilutions from 1 x 10^5) or 4-1BB agonistic antibody (10-fold dilutions from 1 µM) with crosslinker (10-fold dilutions from 2.5 µM), then (**d**) CD4+ and (**e**) CD8+ T cell numbers were measured by flow cytometry, and fold change over PBMC+aCD3 was calculated

PBMC, peripheral blood mononuclear cell; PBS, phosphate-buffered saline; RBC, red blood cell; RCT, red cell therapeutic

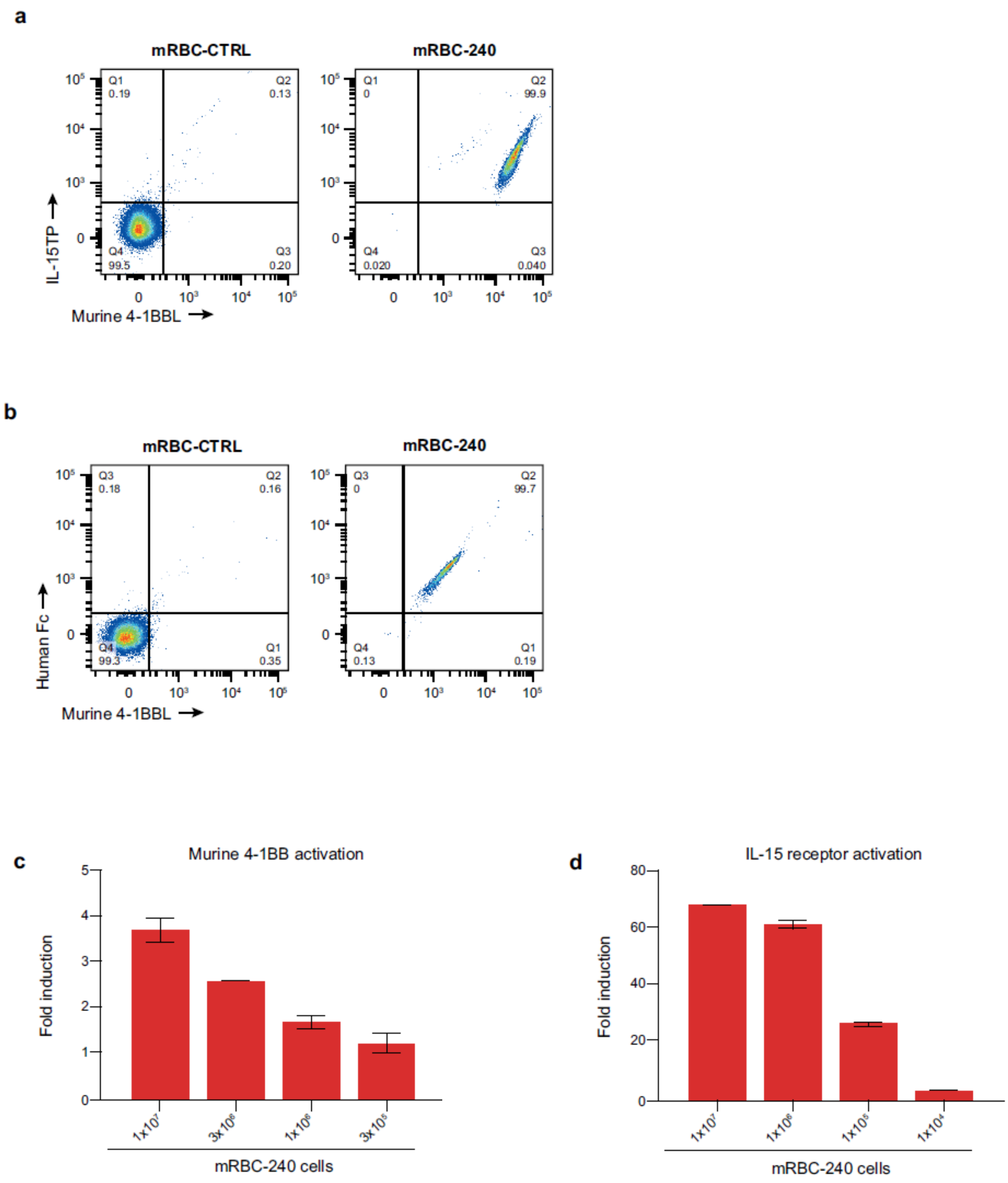
a
mRBC-CTRL
mRBC-240
Q1 0.19
Q2 0.13
Q4 99.5
Q3 0.20
Q1 0
Q2 99.9
Q4 0.020
Q3 0.040
IL-15TP
Murine 4-1BBL
b
mRBC-CTRL
mRBC-240
Q3 0.18
Q2 0.16
Q4 99.3
Q1 0.35
Q3 0
Q2 99.7
Q4 0.13
Q1 0.19
Human Fc
Murine 4-1BBL
c
Murine 4-1BB activation
Fold induction
mRBC-240 cells
d
IL-15 receptor activation
Fold induction
mRBC-240 cells

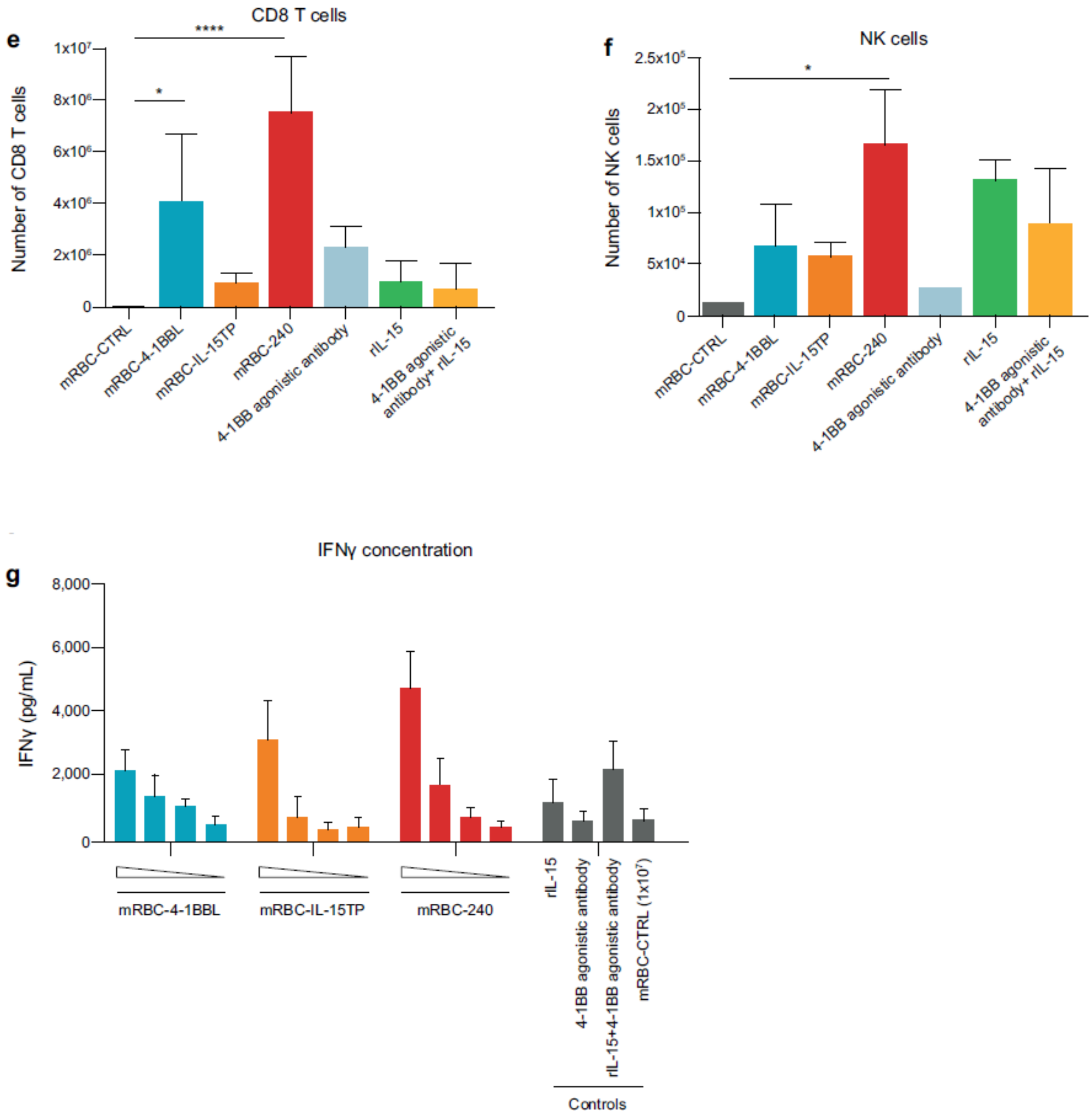

Supplementary figure 2: mRBC-240 engages 4-1BB and IL-15 receptor and activates CD8+ T cells and NK cells in vitro

a mRBC-CTRL or mRBC-240 were stained with anti-mouse 4-1BB Ligand and anti-mouse IgG2a antibodies (IL-15 detection) and expression of 4-1BBL and IL-15TP was determined by flow cytometry. **b** mRBC-CTRL or mRBC-240 were incubated with 1 µg of recombinant human Fc-tagged mouse 4-1BB receptor and binding was detected using anti-mouse IgG2a antibody to

detect mRBC-240 and anti-human Fc antibody to detect the mouse 4-1BB receptor. **c** 3T3 cells expressing 4-1BB receptor were incubated with mRBC-240 for 24 hours and a fold change of the luminescence signal over mRBC-CTRL is represented. **d** HEK-Blue IL-2 SEAP cells were incubated mRBC-240 for 20 hours and a fold change of the luminescence signal over mRBC-CTRL is represented. The number of (**e**) CD8+T cells or (**f**) NK cells following a 4-day stimulation of mouse splenocytes with mRBC-CTRL, mRBC-4-1BBL, mRBC-IL-15TP or mRBC-240 in the presence of 1 µg/mL of anti-CD3. 4-1BB agonistic antibody (10 µg/mL) or recombinant (r) IL-15 (100 ng/mL) are used as controls. Comparisons were analyzed by a one-way ANOVA and compared with mRBC-CTRL and showing as *, $p<0.05$, ****, $p<0.0001$. **g** The concentration of IFNγ (measured by ELISA) in the supernatant after a 24-hour co-culture of mouse splenocytes with either mRBC-4-1BBL, mRBC-IL-15TP,mRBC-240 or controls, in the presence of 2 µg/mL anti-CD3. 4-1BB agonistic antibody (10 µg/mL) and rIL-15 (100 ng/mL) were used as controls. Bars indicate (**c–d**) SD of 2–3 technical replicates and (**e–g**) SD of 3 biological replicates

IFNγ, interferon-γ; IgG, immunoglobulin G; IL-15TP, trans-presented interleukin 15; NK, natural killer; RBC, red blood cell; SD, standard deviation

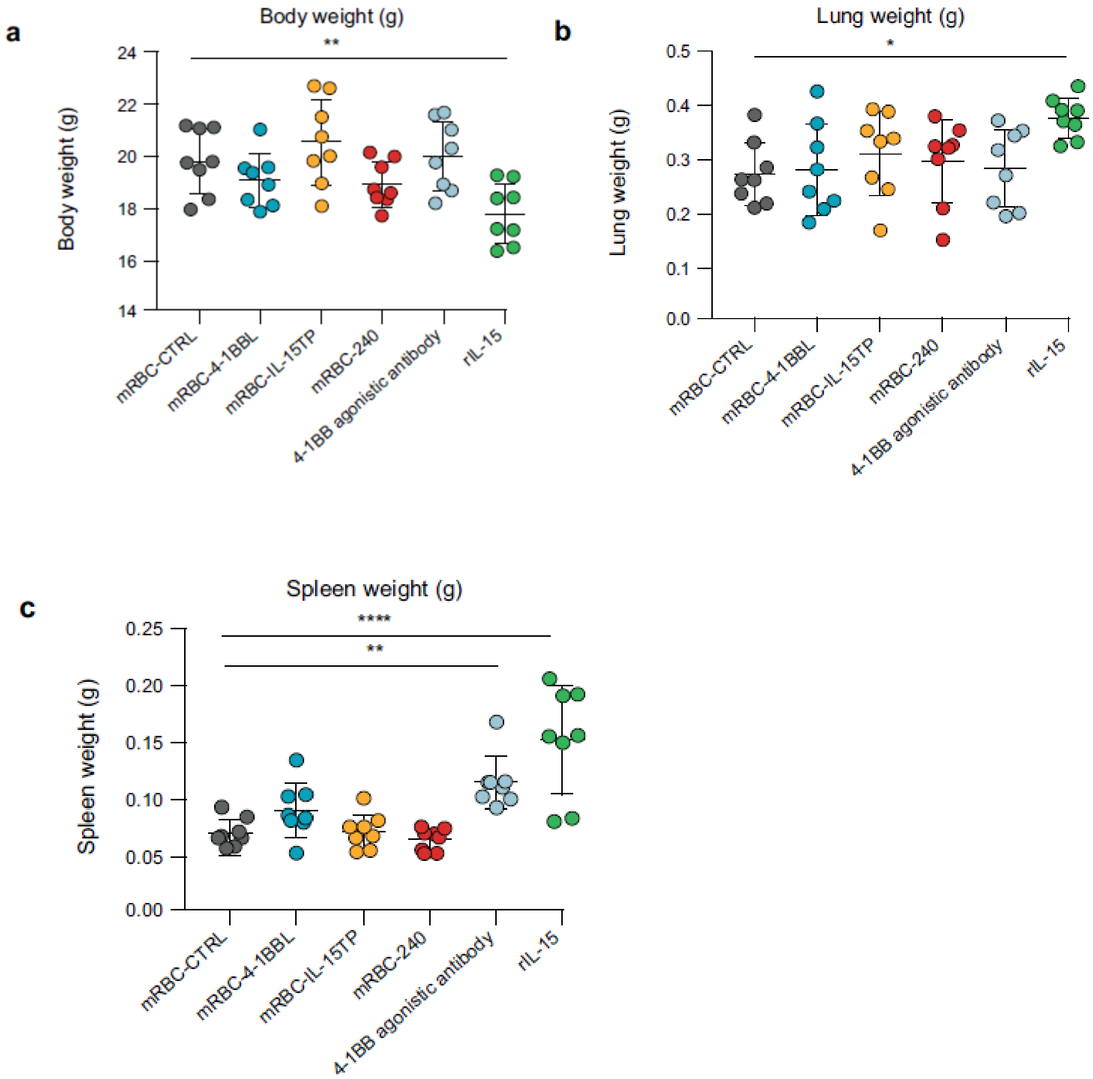

Supplementary figure 3: No changes in organ weights in mice following mRBC-240 treatment

C57BL/6 mice were inoculated intravenously with B16-F10 tumor cells and treated with 1 x 10^9 of either mRBC-4–1BBL, mRBC–IL-15TP or mRBC-240 intravenously or with 2.5 mg/kg of 4-1BB agonistic antibody or 0.2 mg/kg of rIL-15 intraperitoneally on days 1, 5 and 8 post-inoculation. (**a**) Body weight, (**b**) lung weight and (**c**) spleen weight were determined on day 14. Data presented ± SD. All comparisons were analyzed by one-way ANOVA compared to mRBC-CTRL and showing as *, *p*<0.05, **, *p*<0.01, ****, *p*<0.0001

RBC, red blood cell; rIL-15, recombinant interleukin-15; SD, standard deviation

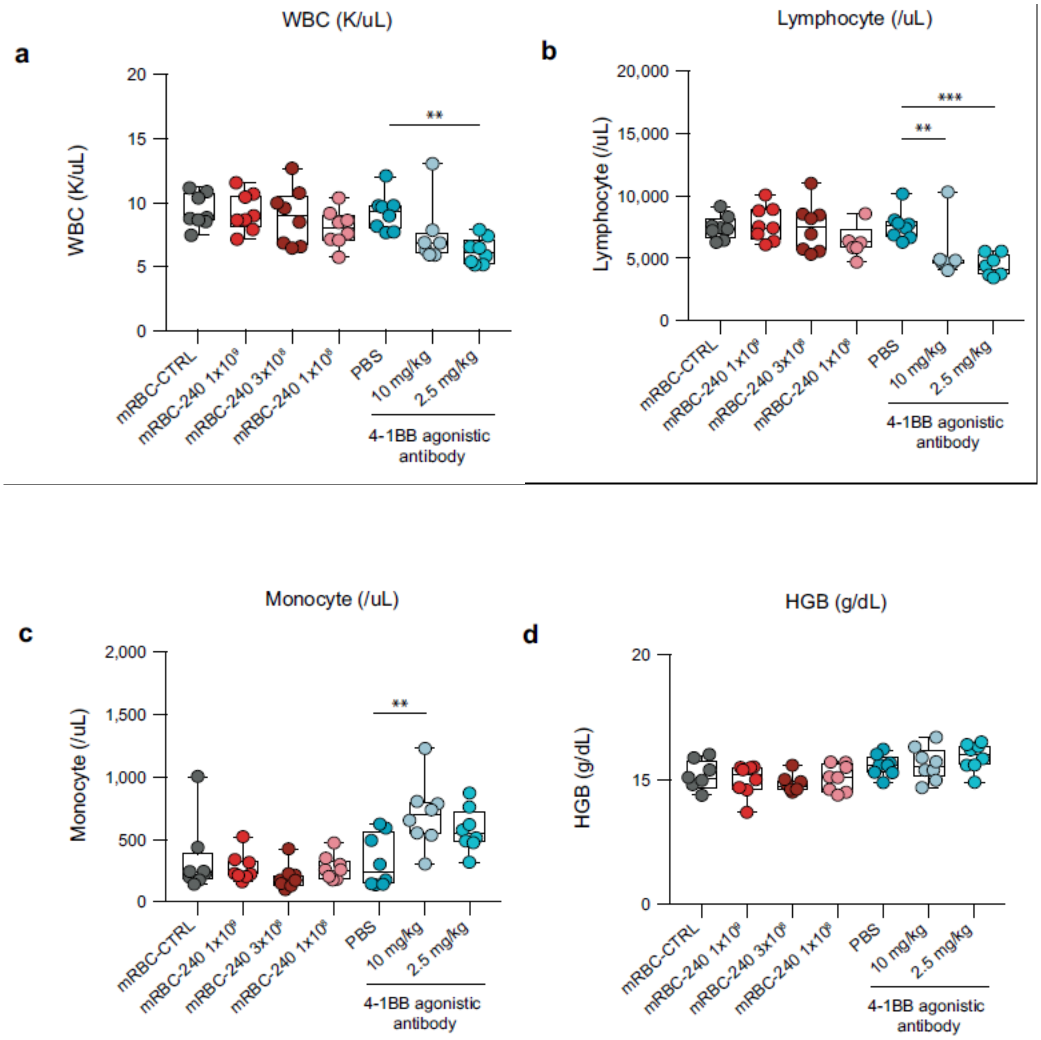

Supplementary figure 4: Complete blood count in mRBC-240-treated mice

The counts of (**a**) white blood cells (K/µL), (**b**) lymphocyte (per µL), (**c**) monocyte (per µL) and (**d**) hemoglobin (g/dL) were analyzed in the blood of C57BL/6 wild-type mice on day 18 following 4 doses of either mRBC-240 (1 x 10^9, 3 x 10^8 or 1 x 10^8), mRBC-CTRL or 4-1BB agonistic antibody (10 mg/kg or 2.5 mg/kg) (*n*=8 mice/group). All comparisons were analyzed by one-way ANOVA and showing as **, p<0.01, ***, p<0.001

HGB, hemoglobin; PBS, phosphate-buffered saline; RBC, red blood cell; WBC, white blood cell
